# Survival-related indicators ALOX12B and SPRR1A are associated with DNA damage repair and tumor microenvironment status in HPV 16-negative head and neck squamous cell carcinoma patients

**DOI:** 10.1186/s12885-022-09722-x

**Published:** 2022-06-29

**Authors:** Jing Li, Ling-Long Tang, Jun Ma

**Affiliations:** 1 Department of Radiation Oncology, Sun Yat-sen University Cancer Center, 651 Dongfeng Road East, Guangzhou, 510060 Guangdong China; 2 State Key Laboratory of Oncology in South China, Collaborative Innovation Center for Cancer Medicine, 651 Dong feng Road East, Guangzhou, 510060 China

**Keywords:** Cancer-associated fibroblasts, DNA damage response, Human papilloma virus 16-negative, Head and neck squamous cell carcinoma, Tumor microenvironment

## Abstract

**Objectives:**

To investigate prognostic-related gene signature based on DNA damage repair and tumor microenvironment statue in human papillomavirus 16 negative (HPV16-) head and neck squamous cell carcinoma (HNSCC).

**Methods:**

For the RNA-sequence matrix in HPV16- HNSCC in the Cancer Genome Atlas (TCGA) cohort, the DNA damage response (DDR) and tumor microenvironment (TM) status of each patient sample was estimated by using the ssGSEA algorithm. Through bioinformatics analysis in DDR_high/TM_high (*n* = 311) and DDR_high/TM_low (*n* = 53) groups, a survival-related gene signature was selected in the TCGA cohort. Two independent external validation cohorts (GSE65858 (*n* = 210) and GSE41613 (*n* = 97)) with HPV16- HNSCC patients validated the gene signature. Correlations among the clinical-related hub differentially expressed genes (DEGs) and infiltrated immunocytes were explored with the TIMER2.0 server. Drug screening based on hub DEGs was performed using the CellMiner and GSCALite databases. The loss-of-function studies were used to evaluate the effect of screened survival-related gene on the motility of HPV- HNSCC cells in vitro.

**Results:**

A high DDR level (*P* = 0.025) and low TM score (*P* = 0.012) were independent risk factors for HPV16- HNSCC. Downregulated expression of ALOX12B or SPRR1A was associated with poor survival rate and advanced cancer stages. The pathway enrichment analysis showed the DDR_high/TM_low samples were enriched in glycosphingolipid biosynthesis-lacto and neolacto series, glutathione metabolism, platinum drug resistance, and ferroptosis pathways, while the DDR_high/TM_low samples were enriched in Th17 cell differentiation, Neutrophil extracellular trap formation, PD − L1 expression and PD − 1 checkpoint pathway in cancer. Notably, the expression of ALOX12B and SPRR1A were negatively correlated with cancer-associated fibroblasts (CAFs) infiltration and CAFs downstream effectors. Sensitivity to specific chemotherapy regimens can be derived from gene expressions. In addition, ALOX12B and SPRR1A expression was associated with the mRNA expression of insulin like growth factor 1 receptor (IGF1R), AKT serine/threonine kinase 1 (AKT1), mammalian target of rapamycin (MTOR), and eukaryotic translation initiation factor 4E binding protein 1 (EIF4EBP1) in HPV negative HNSCC. Down-regulation of ALOX12B promoted HPV- HNSCC cells migration and invasion in vitro.

**Conclusions:**

ALOX12B and SPRR1A served as a gene signature for overall survival in HPV16- HNSCC patients, and correlated with the amount of infiltrated CAFs. The specific drug pattern was determined by the gene signature.

**Supplementary Information:**

The online version contains supplementary material available at 10.1186/s12885-022-09722-x.

## Introduction

Head and neck squamous cell carcinoma (HNSCC) are heterogeneous epithelial tumor that arise from the oropharynx, oral cavity, lip, larynx, nasopharynx, and hypopharynx. The 5-year overall survival rate for patients with HNSCC is approximately 50% [1]. The main causative risk factors include excessive tobacco usage, heavy alcohol usage, and human papillomavirus 16 (HPV16) infection [2]. HPV16 infection-related E6/E7 is generally considered the oncogenic protein associated with HNSCC [3]. HPV-negative (HPV-) HNSCC patients are characterized by distinct molecular landscapes, worse overall survival outcomes and a poor response rate to induction chemotherapy when compared with HPV-positive (HPV+) HNSCC patients [4–6]. Hence, there is an urgent need to investigate the molecular spectra of HPV16 negative (HPV16-) HNSCC and identify survival-related biomarkers.

Tumor cells respond to endogenous or exogenous DNA damage through the DNA damage response (DDR) pathways. Homologous recombination repair (HRR), nonhomologous end joining (NHEJ), mismatch repair (MMR), nucleotide excision repair (NER), and base excision repair (BER) are key pathways participating in the DNA damage response (DDR) [7]. A previous study developed a homologous recombination deficiency (HRD) score at transcriptome level to predict the prognosis of HNSCC patients, and the HRD score was not an independent indicator for prognosis in univariate survival analysis [8]. However, another study has shown that HNSCC patients exhibited double-strand breaks repair (DBR)- and MMR- related genes upregulated as compared with healthy subjects, and patients with low expression of NER-related genes showed prolonged progression-free survival under concurrent chemoradiotherapy treatment [9]. Abnormal DDR status is also an important biological factor leading to radiotherapy resistance [10]. Meanwhile, DDR status could affect expressions of Immune checkpoint protein, interferon receptors, and neoantigens, and thereby affect therapeutic effect of immunotherapy [11, 12]. Therefore, DDR level represents the tumorigenesis and development process, as well as reflecting the efficacy of anti-tumor therapy to a certain extent. Considering the higher DNA repair gene/protein expression levels in HPV+ HNSCC tissue samples than those in HPV- HNSCC samples [13], the prognostic value and therapeutic efficacy value of DDR levels in HPV16- HNSCC patients need to be further clarified. In addition to the DDR level reflecting the malignant evolution of tumor cells, the tumor microenvironment (TM) around tumor cells also affects or reflects the malignant behavior of tumor cells. The TM comprises a network of interactions among blood vessels, immune cells, fibroblasts, adipocyte, endothelial cells, tumor cells, and the surrounding extracellular components [14]. According to previous studies, TM is crucial for modulating tumor progress and evaluating anti-tumor treatment responses [15, 16]. The immune profiles of HPV+ HNSCC is distinct from that in HPV- HNSCC [17, 18]. Interesting, HPV+ patients exhibited enhanced immune cell infiltration compared with HPV- patients [18], which suggests that in the study of analysing the prognostic and efficacy prediction value of tumor microenvironment for HNSCC patients, the HPV infection status of the patients also needs to be considered.

Recently, DNA-targeted therapy combined with radiotherapy, chemotherapy or immunotherapy has shown synergistic anti-tumor effects [19–22]. An earlier study identified prognostic related immune genes based on differences in immune status between tumor tissue and normal tissue in HNSCC patients, which could help develop therapeutic regimens toward specific targets [23]. However, the DDR status differed between tumors and adjacent normal tissue, and immune status (eg, PD-L1 expression) in tumor tissue varies among patients. In the present study, we used the TCGA and GEO databases to explore and verify prognostic-related genes reflecting both the DDR and the TM status as well as exploring the drug sensitivity pattern in HPV16- HNSCC patients.

## Materials and methods

### Patient cohort and data source

The RNA sequencing data (fragments per kilobase per million (FPKM) and read counts) for HNSCC were downloaded from the Cancer Genome Atlas (TCGA) database (https://portal.gdc.cancer.gov). Patient clinical data were obtained from the Broad Genome Data Analysis Centers Firehose server (https://gdac.broadinstitute.org). Related survival data were downloaded from the UCSC Xena browser (https://xenabrowser.net). Based on the HPV status recorded in the Firehose server and available survival data in the Xena browser, 433 HPV16- HNSCC patients were included in the TCGA discovery cohort. The validation cohort included 210 HPV16- patients in the Gene Expression Omnibus (GEO, https://www.ncbi.nlm.nih.gov/geo) database (GSE65858 data set) and 97 HPV- patients in the GSE41613 data set. The data in this study were all obtained from open available databases, and data download process comply with the data use certification agreement of TCGA, Firehose, UCSC Xena and GEO databases. The requirements for institutional review board approval and informed consent were waived.

### Collection of DDR-related gene sets and cluster TCGA samples at the DNA damage repair level

Five DDR-associated gene sets were collected from online Molecular Signatures Database (MSigDB) (https://gsea-msigdb.org). There were 55 genes in the HRR gene set (hsa03440, R-HSA-5693579), 13 in the NHEJ gene set (hsa03450), 23 in the MMR gene set (R-HSA-5358508, hsa03430), 111 in the NER gene set (hsa03420, R-HSA-5696398), and 98 in the BER gene set (hsa03410, R-HSA-73884). The single-sample Gene Set Enrichment Analysis (ssGSEA) algorithm (R package “GSVA”) was used to obtain a score for the DDR level in each HPV16- tumor sample (FPKM) of the TCGA discovery cohort. The whole cohort was then clustered into DDR_high and DDR_low groups via the “sparcl” package in R [24, 25]. In univariate survival analysis, the log-rank test was used to compare the difference in overall survival rate between the above two groups.

### Tumor microenvironment status identification in the TCGA cohort

In the TCGA discovery cohort, the ESTIMATE algorithm (R package “ESTIMATE”) was used to calculate scores for immune and stromal cell infiltration in the transcriptome profiles (FPKM) [26]. The maximally selected rank statistics method (“maxstat” package in R) was applied to classify the cohort into tumor microenvironment high (TM_high) and tumor microenvironment low (TM_low) groups [27]. Among the two groups, the TM_high group contains more immune and stromal cell infiltration than that of the TM_low group. The prognostic value of the TM classification method was calculated using the Kaplan-Meier curve and log-rank tests. Combining DDR and TM status in TCGA discovery cohort can obtain four subgroups, namely, The DDR_high/TM_low, DDR_high/TM_high, DDR_low/TM_high, and DDR_low/TM_low groups. The DDR_high/TM_high and DDR_high/TM_low groups were extracted and integrated into the TCGA final discovery cohort. A multivariate survival analysis was conducted to evaluate the influence of the grouping factor on the overall survival rate for patients in the TCGA final discovery cohort.

### Somatic alteration analysis of the DDR_high/TM_high and DDR_high/TM_low groups

Gene mutation data were obtained from the TCGA database (https://portal.gdc.cancer.gov). The “maftool” package in R was applied to visualize the top 20 mutant genes in DDR_high/TM_low and DDR_high/TM_high groups, respectively [28]. A forest plot showed the mutant genes that significantly differed between the above two groups (*P* < 0.05).

### Extracted DDR- and TM-related hub genes and enrichment analysis of the hub genes in the TCGA final discovery cohort

For the TCGA final discovery cohort, the raw data (read counts) for tumor samples were standardized using the cpm function and genes with high expression remained (mean read counts per million was larger than one). Differentially expressed genes (DEGs) with FDR < 0.05 and log |fold change| > 0.5 between DDR_high/TM_high and DDR_high/TM_low groups were calculated with the “limma” package in R. A weighted gene co-expression network analysis (WGCNA) algorithm (“WGCNA” package in R) was used to select the DEGs modules correlated with the DDR_high/TM_high and DDR_high/TM_low groups [29]. In the WGCNA analyzing process, DEGs with variance > 50% among samples were selected. Using pearson’s correlation coefficient, paired genes were used to build a co-expression network. The co-expression network was transformed into an adjacency matrix by selecting the soft threshold (R^2^ > 0.8). Then, a topology overlap matrix (TOM) was established using the tomsimilarity function to calculate the degree of association of genes in the adjacency matrix. The distance matrix 1-TOM was used to construct a hierarchical cluster tree and identify the various modules via dynamic tree cut. Then, modules with optimal eigenvalue similarity values were extracted for further analysis. Finally, a plot was constructed to show the correlation between the extracted modules and the subgroups. Eigenvalue gene modules that were significantly correlated with subgrouping (*P* < 0.05) were extracted for further analysis. Hub genes were obtained by applying a protein–protein interaction (PPI) analysis to the eigenvalue gene modules (STRING (http://string-db.org)). For the nodule degree rank, the top 50 or 100 genes were defined as hub genes. Enrichment analysis of the hub genes in each eigenvalue gene module was performed with the “clusterProfiler” package in R [30], and the Kyoto Encyclopedia of Genes and Genomes (KEGG) pathways analysis in the “clusterProfiler” package is based on KEGG website (https://www.kegg.jp/) [31–33]. *P* < 0.05 was considered statistically significant.

### Prognostic-related hub gene identification and validation

A univariate survival analysis (*P* < 0.2) was conducted to select the significant hub genes related to overall survival rate of patients in the TCGA final discovery cohort. Independent prognostic-related hub genes were identified by multivariate survival analysis. A correlation analysis between the prognostic-related hub genes and the clinical traits was performed. The prognostic-related hub genes were validated in the GSE65858 and GSE41613 data sets.

### Therapeutic response prediction

Immunophenoscores (IPS) were calculated according to the four major immunogenicity categories, namely, effector cells, immunosuppressive cells, MHC molecules, and immunomodulators [34]. The Cancer Immunome Atlas (TCIA; https://tcia.at/home) webtool provided four indexes for each TCGA patient: 1, The IPS index, and a high IPS value showed increased immunogenicity; 2, The IPS-PD1/PD-L1/PD-L2 blocker index, and a high value means more sensitivity to PD1/PD-L1/PD-L2 antibodies; 3, The IPS-CTLA4 blocker index, and a high value means more sensitivity to CTLA4 antibodies; 4, The IPS-PD1/PD-L1/PD-L2 + CTLA4 blocker index, and a high value means more sensitivity to PD1/PD-L1/PD-L2 and CTLA4 antibodies. According to the expression of hub genes, samples were divided into hub gene high- and low- group by the maximally selected rank statistics method. IPS and derived indexes were downloaded, and the differences in those indexes between high- and low- groups were analysed. Except for surgery and radiotherapy, other important treatment options for HNSCC patients are chemotherapy and small-molecule targeted drugs. At the same time, we are gradually increasing our understanding of the rationality of concurrent chemotherapy and immunotherapy. The CellMiner (https://discover.nci.nih.gov/cellminer) and GSCALite (http://bioinfo.life.hust.edu.cn/web/GSCALite/) databases can provide correlations between specific genes and drug sensitivity in the NCI-60 cell line set and in the CTRP or GDSC databases, respectively. Accordingly, we analysed the associations between the hub genes and the drug response in the CellMiner, CTRP, and GDSC databases.

### Correlations among prognostic-related hub genes, immune infiltration levels, and downstream immune cell effectors

The online webserver TIMER2.0 database (http://timer.comp-genomics.org) is a comprehensive resource providing gene-associated immune infiltration data across 32 cancer types. TIMER2.0 database can provide multiple algorithms such as xCell, CIBERSORT, EPIC, MCP-counter and TIMER algorithms. The relationships between prognostic-related hub genes and infiltrated immune cells were explored. A Spearman’s rank analysis was performed to analyse the correlations between the hub genes and the downstream immune cell effectors. The associate between the mRNA expression of the identified hub genes and the mRNA expression of insulin like growth factor 1 receptor (IGF1R), AKT serine/threonine kinase 1 (AKT1), mammalian target of rapamycin (MTOR), and eukaryotic translation initiation factor 4E binding protein 1 (EIF4EBP1) for HPV- HNSCC were also explored in TIMER2.0 database.

### Cell culture and transfection of RNA oligonucleotides

HPV- HNSCC cell lines (HN6, CAL27) were cultured in DMEM with 5% FBS (Gibco, USA) and maintained in a humidified 5% CO2 environment at 37 °C. Total RNA was extracted using RNAiso Plus (Takara 9109, Japan). Quantitative Real-time PCR (qRT-PCR) was performed with ABI Real-Time PCR System (ABI 7500, Thermofisher CA). Glyceraldehyde 3-phosphate dehydrogenase (GAPDH) was used to normalize the mRNA expression of test gene, and the ∆∆ Ct method was conducted to calculate the relative expression levels of test gene. Test gene primers used in qRT-PCR were listed as follows: ALOX12B, forward, 5′-TCTCACTGACCATTGTGGGGA-3′; ALOX12B, reverse, 5′-TTGTGCAGGCGGATGATGATG-3′. The small interfering RNA (siRNA) was chemically synthesized by Geneseed (Guangzhou, China). HPV- HNSCC cell lines was transfected with siRNA using lipofectamine™ 2000 (Invitrogen, USA). The siRNA-mediated knockdown of ALOX12B was achieved by targeting the sequence 5′-CGCTATGCGGAGTTCTACA-3′.

### Cell proliferation, invasion assays and metastasis assays

The CCK8 assays (YEASEN, Shanghai) was used to assess cell proliferation. HN6 cells were seed into 96-well plates with 1 × 10^3^/well and Cell Counting Kit-8 solution (YEASEN, Shanghai) was added to each well. Subsequently, the cells counting was performed daily for 4 days. The matrigel-coated transwell assay and transwell migration assay were used to test invasion and migration ability, respectively. In the matrigel-coated transwell assay, HN6 cells (1 × 10^5^/well) were seed into 6-well transwell plates (COSTAR, USA) that precoated Matrigel solution (BD Biosciences, USA), and the 6-well plates was incubated in a humidified incubator with 5% CO2 at 37 °C for 24 h. In the transwell migration assay, HN6 cells (1 × 10^5^/well) were seed into 6-well transwell plates (COSTAR, USA), and tested after 24 h. The migrated or invaded cells were fixed and stained with crystal violet and counted using ImageJ software.

### Statistical analysis

All data were processed in R v. 3.6.1 (http://www.R-project.org) and GraphPad Prism 8 software (GraphPad Software, Inc., USA). *P* < 0.05 was considered statistically significant in both the multivariate survival analysis and correlation analysis.

## Results

### DDR-related patients clustering in the TCGA discovery cohort

**Fig. 1:**
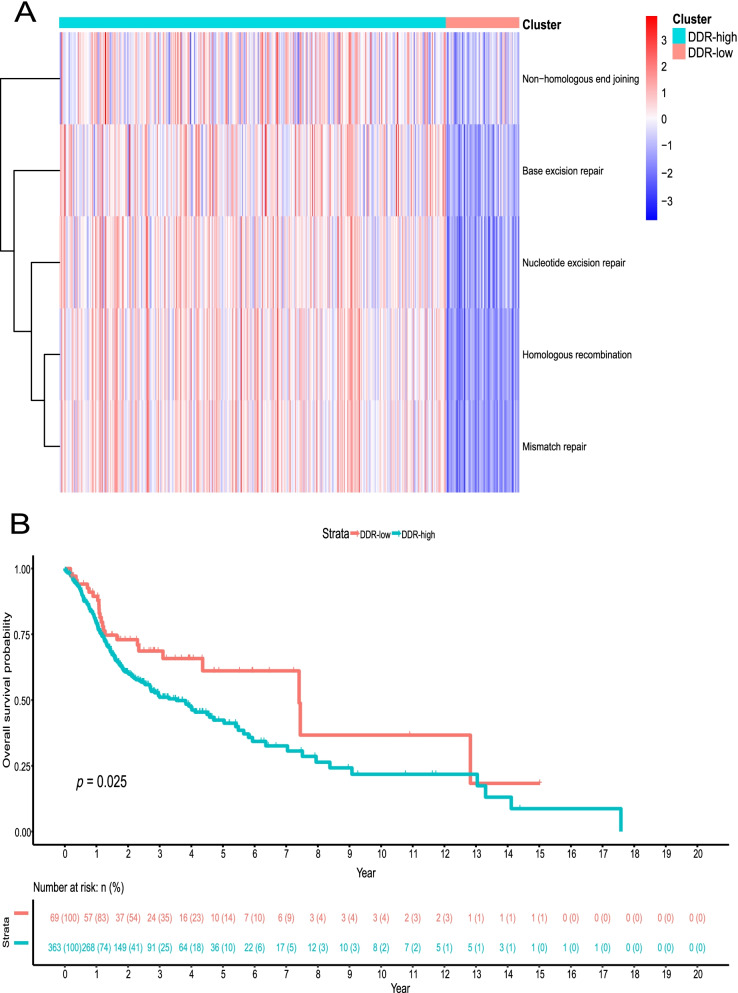
Patient sample stratification based on DNA damage response (DDR) level in human papillomavirus 16 negative (HPV16-) head and neck squamous cell carcinoma (HNSCC) TCGA discovery cohort (*n* = 433). **A** ssGSEA matrix plot of two subtypes identified in HPV16- HNSCC TCGA discovery cohort according to five DDR-associated genesets. **B** Kaplan-Meier (K-M) plot of overall survival probability for patients in the above two subtypes. One patient belonging to DDR_high subtype lacked survival data as shown in risk table

A total of 433 HPV16- HNSCC patients were included in the TCGA discovery cohort (Table S1). Based on the DDR-related gene sets (Table S2), the ssGSEA method categorized patients into the DDR_high group (Cluster 1; 364 patients) and the DDR_low group (Cluster 2; 69 patients) (Fig. 1A). The log-rank test showed a difference in survival between the two groups (*P* = 0.025) (Fig. 1B).

### TM- and DDR- status classification in the TCGA discovery cohort

**Fig. 2:**
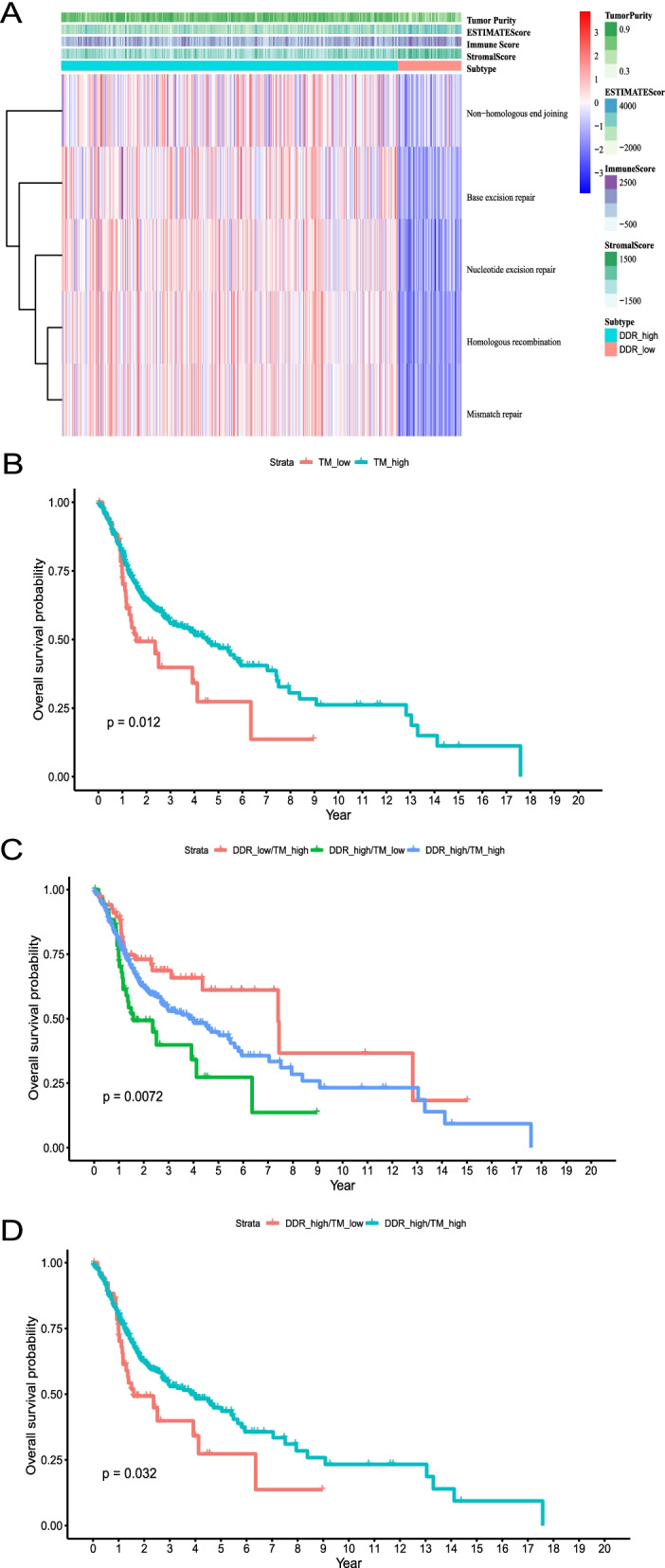
The Kaplan–Meier (K-M) overall survival (OS) curve of patients in the TCGA final discovery cohort (*n* = 364). **A** Two clusters were obtained from TCGA discovery cohort (*n* = 433) by dichotomizing tumor microenvironment (TM) score (“ESTIMATE” package in R). **B** K-M plot of OS probability in high- and low- TM score group. **C** K-M plot of OS probability in DDR_high/TM_low, DDR_high/TM_high, and DDR_low/TM_high groups. **D** K-M plot of OS probability for patients in the TCGA final discovery cohort including only DDR_high/TM_high and DDR_high/TM_low groups. One patient in the TCGA final discovery cohort lacked survival data as mentioned in Fig. 1

In the TCGA discovery cohort, we divided patients into two cluster through ESTIMATE scoring and the maximally selected rank statistics method. The cut-off value for the maximally selected rank statistics algorithm was − 951.85 (Fig. 2A). A univariate survival analysis showed a significant difference in survival between TM_high (*n* = 380) and TM_low (*n* = 53) groups (*P* = 0.012; Fig. 2B). After integrating the DDR-related clustering, there were 53, 311, 69, and 0 patients in the DDR_high/TM_low, DDR_high/TM_high, DDR_low/TM_high and DDR_low/TM_low groups, respectively. We performed a survival log-rank test among DDR_high/TM_low, DDR_high/TM_high, and DDR_low/TM_high groups and found that the DDR_low/TM_high group showed the best overall survival rate (*P* = 0.0072; Fig. 2C). We compared the survival rates of the DDR_high/TM_high and DDR_high/TM_low groups and observed that the former exhibited higher survival than the latter (*P* = 0.032; Fig. 2D). Then, we integrated the data for the DDR_high/TM_low (*n* = 53) and DDR_high/TM_high (*n* = 311) groups to create the TCGA final discovery cohort (*n* = 364).

The clinical characteristics of the DDR_high/TM_low and DDR_high/TM_high groups were shown in Table S3. The multivariate survival analysis disclosed that the DDR_high/TM_low status was a risk factor for overall survival (OS) in HPV16- HNSCC (Table 1). As shown in Table S3, N stage, T stage, TNM stage, alcohol history, smoking history, lymphovascular invasion status, margin status, perineural invasion status, pathological nodal extracapsular spread status, anatomic neoplasm subdivision, neoadjuvant treatment, radiation therapy, additional pharmaceutical therapy, and additional radiation therapy were all balanced between the DDR_high/TM_low and DDR_high/TM_high groups. However, two groups differed in terms of sex and age.

**Table 1:** Multivariate survival analysis in the TCGA final discovery cohort

Overall survival		HR (95%CI)	***P*** value
T stage	T4 vs. T1–3	0.76 (0.55–1.06)	0.105
N stage	N2–3 vs. N0–1	1.73 (1.22–2.44)	0.002**
Age	> 60 vs. < = 60	1.39 (1.00–1.95)	0.056
Sex	Male vs. Female	0.75 (0.53–1.05)	0.097
Grade category	G3 vs. G1–2	0.94 (0.66–1.35)	0.746
Subgroups	DDR-high/TM-low vs. DDR-high/TM-high	1.78 (1.15–2.75)	0.009**

*HR* Hazard ratio, *CI* Confidence interval, *DDR-high* High DNA repair level at transcriptome level, *TM-high/−low* Tumor microenvironment score -high/−low at transcriptome level, respectively. ** *p* value< 0.01

### Somatic mutant gene distinction between DDR_high/TM_low and DDR_high/TM_high groups in the TCGA final discovery cohort

**Fig. 3:**
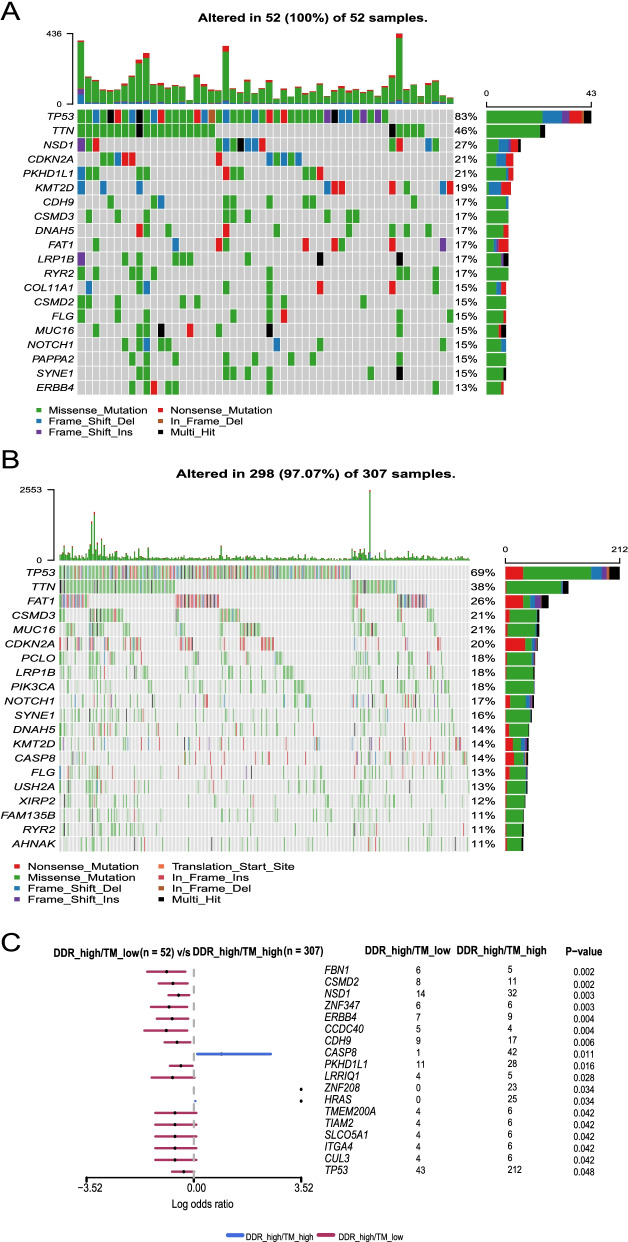
Somatic mutant genes in DDR_high/TM_low and DDR_high/TM_high groups of the TCGA final discovery cohort. **A** Mutant genes (top 20) in DDR_high/TM_low group. **B** Mutant genes (top 20) in DDR_high/TM_high group. **C** Differential mutant genes between DDR_high/TM_low and DDR_high/TM_high groups

We analysed the top 20 genes with the highest mutation frequency in the TCGA final discovery cohort. In the DDR_high/TM_low group, they were TP53, TTN, NSD1, CDKN2A, and PKHD1L1 (Fig. 3A). In the DDR_high/TM_high group, they were TP53, TTN, FAT1, CSMD3, and MUC16 (Fig. 3B). NSD1, CSMD2, ERBB4, ITGA4, CUL3, and TP53 showed relatively a higher mutation rate in the DDR_high/TM_low group, whereas CASP8 showed a relatively higher mutation frequency in the DDR_high/TM_high group (Fig. 3C).

### TM- and DDR- related hub DEGs identification and functional enrichment analysis in the TCGA final discovery cohort

**Fig. 4:**
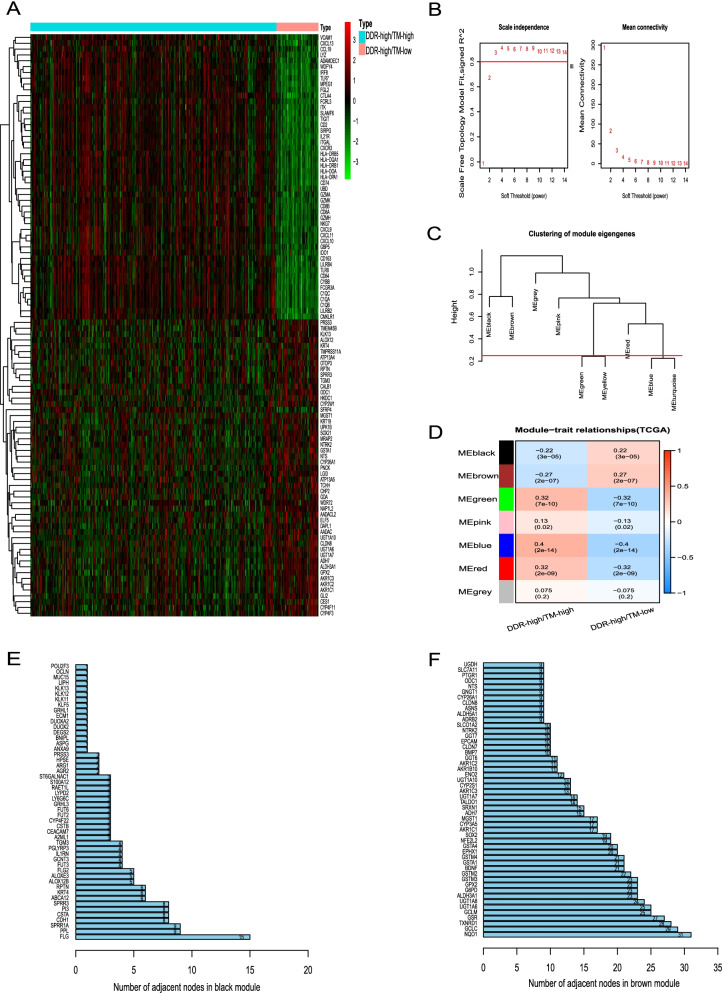
Identification of DDR- and TM- related hub genes in the TCGA final discovery cohort. **A** Heatmap of clustered top 100 differentially expressed genes (DEGs) between DDR_high/TM_high and DDR_high/TM_low groups. **B**-**D** WGCNA algorithm screened out seven eigengenes module (blue, green, red, pink, black, brown, and grey) based on DDR and TM status. **E**-**F** The top 50 hub genes selected in black and brown modules by protein-to-protein network method; respectively

We obtained 2140 DEGs in the TCGA final discovery cohort. Of these, 1589 were downregulated and 551 were upregulated in the DDR_high/TM_low group, when compared with the DDR_high/TM_high group. The heatmap shows clustering of the top 50 upregulated and top 50 downregulated genes in the DDR_high/TM_low group (Fig. 4A). During the WGCNA calculation, the corresponding soft threshold was three and R^2^ > 0.8 (Fig. 4B). Modules with eigenvalue similarity > 0.75 were merged for further analysis (Fig. 4C). Then, the WGCNA algorithm identified seven DEGs modules designated blue, green, red, pink, black, brown, and gray (Fig. 4D). For the PPI analysis, we calculated gene nodes in the black, brown, blue, green, and red gene modules, respectively. After ranking the connecting node numbers between genes in each module, we screened out the top 50 hub genes in the black and brown modules (Fig. 4E, F). Similarly, we screened out the top 100 hub genes in the blue, green, and red modules, respectively (Fig. S1).

**Fig. 5:**
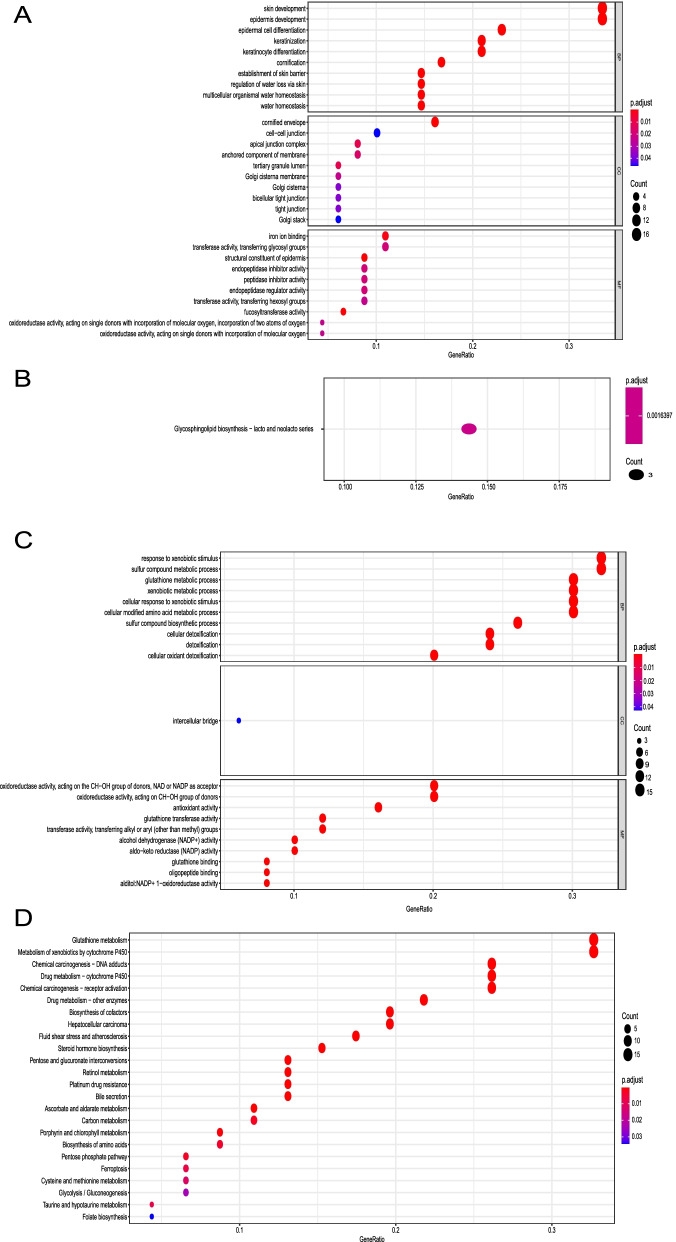
Biological characteristics of hub genes in black and brown modules. **A** Biological process, molecular function, and cellular component terms enriched by hub genes in black module. **B** KEGG pathway enriched by hub genes in black module. **C** Biological process, molecular function, and cellular component terms enriched by hub genes in brown module. **D** KEGG pathways enriched by hub genes in brown module

**Fig. 6:**
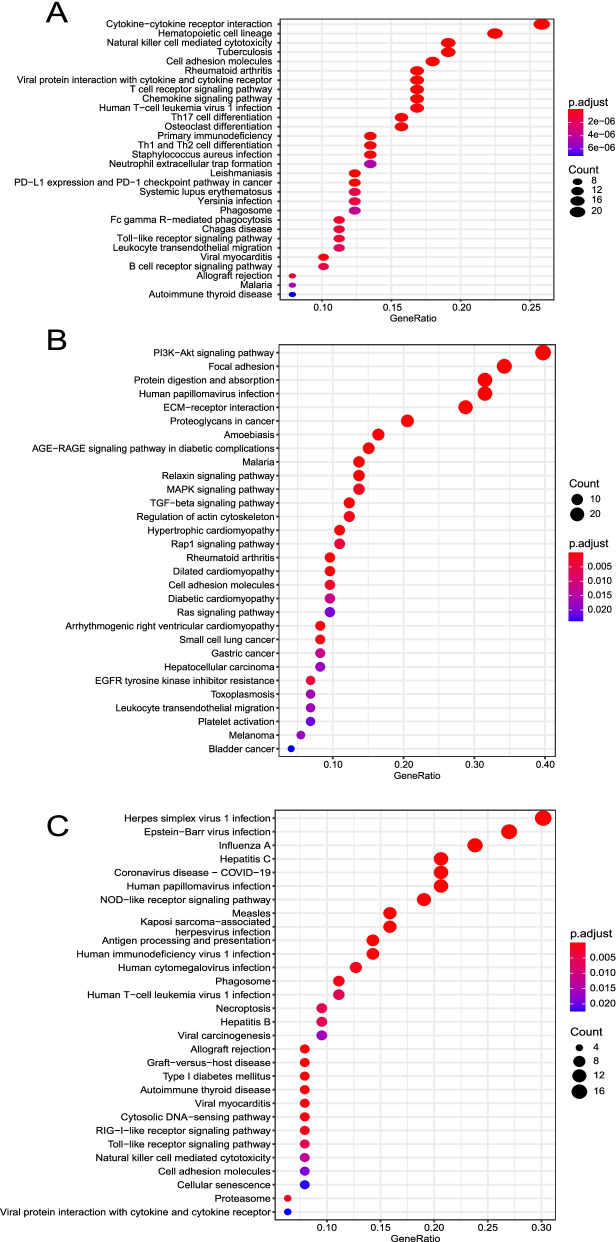
KEGG pathways analysis of hub genes in blue, green, and red modules. **A** KEGG pathways enriched by top 100 hub genes in blue module. **B** KEGG pathways enriched by top 100 hub genes in green module. **C** KEGG pathways enriched by top 100 hub genes in red module

For the black gene module, the top 50 hub genes were enriched in ten biological process (BP) items, ten molecular function (MF) items, ten cellular component (CC) items, and one KEGG pathway (Fig. 5A, B). For the brown module, the top 50 hub genes were enriched in ten biological process (BP) items, ten molecular function (MF) items, one cellular component (CC) item, and 24 KEGG pathways (Fig. 5C, D). In the BP analysis of the black module, the hub genes were enriched in keratinocyte differentiation, epidermal cell differentiation, and keratinization. In the BP analysis of the brown module, the hub genes were enriched in xenobiotic stimulus, glutathione metabolic process, and cellular detoxification. The involved KEGG pathways in the black module were glycosphingolipid biosynthesis-lacto and neolacto series. The KEGG pathways involved in the brown module were metabolism of glutathione metabolism, drug metabolism−cytochrome P450, platinum drug resistance, and ferroptosis. For the blue gene module, the top 100 hub genes were enriched in several KEGG pathway (Fig. 6A), such as, Natural killer cell mediated cytotoxicity, Th17 cell differentiation, Neutrophil extracellular trap formation, PD − L1 expression and PD − 1 checkpoint pathway in cancer, and Leukocyte trans-endothelial migration pathways. For the green module, the top 100 hub genes were enriched in 30 KEGG pathways (Fig. 6B), such as, PI3K − Akt signaling pathway, Focal adhesion, MAPK signaling pathway, and TGF − beta signaling pathways. For the green module, the top 100 hub genes were enriched in 31 KEGG pathways (Fig. 6C), such as, Herpes simplex virus 1 infection, Epstein−Barr virus infection, Human papillomavirus infection, and Viral carcinogenesis pathways.

### Prognostic value of the hub genes in the TCGA final discovery and GEO validation data sets

Among the 100 hub genes in the black and brown modules, 28 genes were correlated with OS in HPV16- HNSCC (Table 2). The multivariate survival analysis identified ALOX12B and SPRR1A as protective survival-related genes (Table 3). Geographical validations were performed on the GSE65858 and GSE41613 data sets. Multivariate survival analysis revealed that SPRR1A was significantly correlated with better OS in both GEO cohorts, whereas ALOX12B was an independent predictor of survival in patients at GSE65858 data set.

**Table 2:** Univariate survival analysis for the prognostic value of hub genes in the TCGA final discovery cohort

Gene	HR	HR.95%Low	HR.95%High	***P*** value
ALOXE3	0.9723	0.9386	1.0073	0.1195
ANXA9	0.9758	0.9420	1.0107	0.1714
FUT2	0.9803	0.9541	1.0072	0.1492
MUC15	1.0238	0.9913	1.0574	0.1535
ASPG	0.9579	0.9044	1.0146	0.1428
PI3	1.0000	0.9999	1.0000	0.1149
CSTA	0.9997	0.9994	1.0001	0.1825
BNIPL	0.9868	0.9682	1.0057	0.1709
HPSE	0.9639	0.9138	1.0167	0.1768
SPRR1A	0.9999	0.9999	1.0000	0.1046
GCNT3	1.0336	1.0042	1.0638	0.0248
LY6G6C	0.9962	0.9909	1.0016	0.1647
PRSS3	0.9933	0.9834	1.0034	0.1913
ECM1	0.9983	0.9959	1.0007	0.1583
ALOX12B	0.9886	0.9777	0.9996	0.0420
PTGR1	1.0040	1.0002	1.0077	0.0383
TXNRD1	1.0033	0.9994	1.0073	0.0997
NQO1	1.0018	0.9995	1.0042	0.1270
ASNS	1.0169	1.0017	1.0324	0.0294
SLC7A11	1.0092	0.9981	1.0205	0.1047
G6PD	1.0019	0.9995	1.0044	0.1215
ENO2	1.0095	0.9973	1.0219	0.1284
SRXN1	1.0311	0.9864	1.0778	0.1753
GNGT1	1.1803	1.0139	1.3740	0.0325
ADH7	1.0047	1.0009	1.0086	0.0162
CLDN8	1.0242	0.9933	1.0560	0.1260
ODC1	0.9994	0.9985	1.0003	0.1863
CYP2S1	1.0039	0.9993	1.0086	0.0984

**Table 3:** Multivariate survival analysis for the prognostic value of SPRR1A and ALOX12B in the TCGA final discovery cohort and GEO validation data sets

Dataset	Stratification	HR (95%CI)	***P*** value
**SPRR1A expression for the TCGA final discovery cohort**			
T stage	T3–4 vs. T1–2	1.17 (0.82–1.67)	0.380
N stage	N2–3 vs. N0–1	1.50 (1.06–2.14)	0.022 *
Age		1.03 (1.01–1.04)	< 0.001 ***
Sex	Male vs. Female	0.84 (0.59–1.20)	0.344
Grade category	G3 vs. G1–2	0.88 (0.61–1.27)	0.486
SPRR1A		0.99 (0.99–1.00)	0.040 *
**SPRR1A expression for GSE65858 data set**			
T stage	T3–4 vs. T1–2	1.92 (1.13–3.26)	0.016 *
N stage	N2–3 vs. N0–1	2.26 (1.38–3.70)	0.001 **
Age		1.03 (1.01–1.05)	0.005**
Sex	Male vs. Female	1.01 (0.55–1.85)	0.972
SPRR1A		0.81 (0.69–0.94)	0.007 **
**SPRR1A expression for GSE41613 data set**			
Cancer stage	Stage III-IV vs. Stage I-II	3.33 (1.68–6.62)	0.001 ***
Age		0.99 (0.97–1.02)	0.726
Sex	Male vs. Female	1.22 (0.67–2.22)	0.511
SPRR1A		0.88 (0.79–0.98)	0.016 *
**ALOX12B expression for the TCGA final discovery cohort**			
T stage	T3–4 vs. T1–2	1.13 (0.79–1.60)	0.506
N stage	N2–3 vs. N0–1	1.52 (1.07–2.16)	0.018 *
Age		1.03 (1.01–1.04)	< 0.001 ***
Sex	Male vs. Female	0.84 (0.59–1.20)	0.335
Grade category	G3 vs. G1–2	0.90 (0.63–1.29)	0.561
ALOX12B		0.99 (0.98–0.99)	0.034 *
**ALOX12B expression for GSE65858 data set**			
T stage	T3–4 vs. T1–2	1.93 (1.13–3.29)	0.016 *
N stage	N2–3 vs. N0–1	2.16 (1.32–3.55)	0.002 **
Age		1.03 (1.01–1.06)	0.002**
Sex	Male vs. Female	1.12 (0.62–2.05)	0.707
ALOX12B		0.76 (0.59–0.98)	0.032 *
**ALOX12B expression for GSE41613 data set**			
Cancer stage	Stage III-IV vs. Stage I-II	3.52 (1.78–6.96)	< 0.001 ***
Age		0.99 (0.97–1.02)	0.895
Sex	Male vs. Female	1.20 (0.66–2.20)	0.555
ALOX12B		0.93 (0.83–1.04)	0.209

*HR* Hazard ratio, *CI* Confidence interval, *SPRR1A* Small Proline Rich Protein 1A, *ALOX12B* Arachidonate 12-Lipoxygenase, 12R Type. * *p* value< 0.05; **p value< 0.01; ***p value< 0.001

**Fig. 7:**
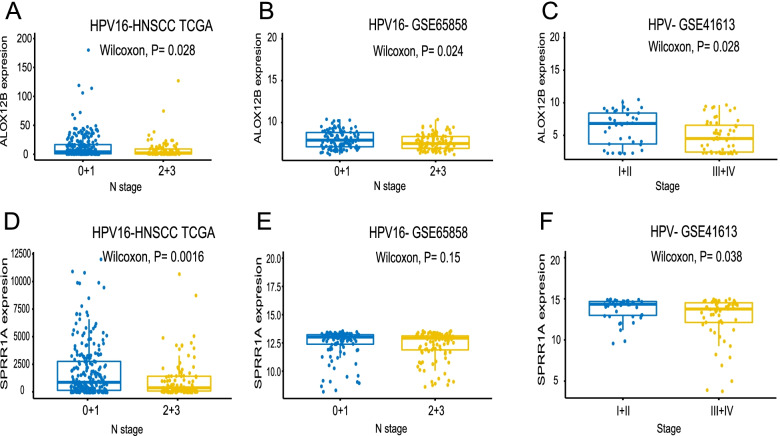
Wilcoxon rank test of prognostic-related hub gene expression between early and advanced TNM stage groups. **A**-**C** Correlation between the expression of ALOX12B and N stage in the TCGA final discovery cohort and GSE65858 data set, respectively. Association between the expression of ALOX12B and TNM stage in GSE41613 data set. **D**-**F** Correlation between the expression of SPRR1A and N stage in the TCGA final discovery cohort and GSE65858 data set, respectively. Association between the expression of SPRR1A and TNM stage in GSE41613 data set

Correlation analysis demonstrated that ALOX12B was upregulated in N0–1 stage as compared with N2–3 stage in the TCGA final discovery cohort (*P* = 0.028). Elevated ALOX12B expression was also observed in the earlier cancer stage in the GSE65858 and GSE41613 data sets (*P* = 0.024 and *P* = 0.028, respectively). SPRR1A downregulation was correlated with advanced N stage and cancer stage in the TCGA final discovery cohort (*P* = 0.0016) and GSE41613 (*P* = 0.038), respectively (Fig. 7).

### Drug sensitivity analysis based on prognostic gene expression levels

**Fig. 8:**
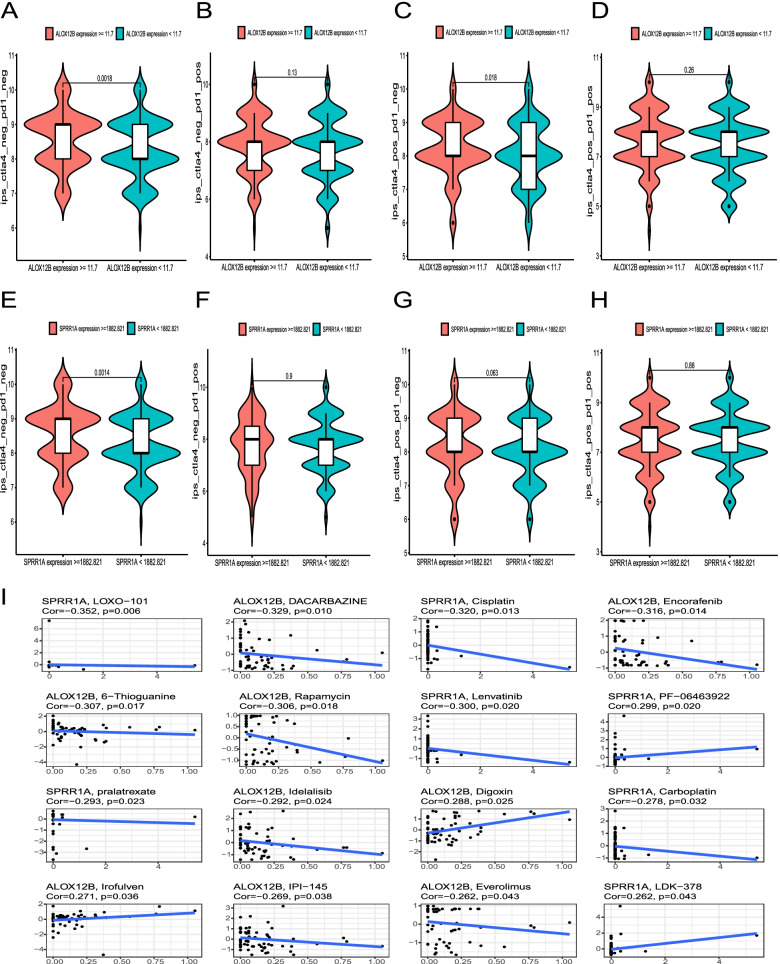
Immunogenicity analysis based on the expression of prognostic-related hub genes. Differences of IPS, IPS-PD1/PD-L1/PD-L2 blocker, IPS-CTLA4 blocker, and IPS-PD1/PD-L1/PD-L2 + CTLA4 blocker between the groups with high and low expression of ALOX12B (**A**-**D**) and SPRR1A (**E**-**H**), respectively. **I** Drug patterns based on ALOX12B and SPRR1A in CellMiner database, and panels were arranged according to correlation coefficient

Increased IPS and IPS-CTLA4 blocker indexes were observed in the groups with high expression of ALOX12B (*P* = 0.0018, *P* = 0.018) or SPRR1A (*P* = 0.0014, *P* = 0.063) (Fig. 8A–H). The IPS-PD1/PD-L1/PD-L2 blocker and IPS-PD1/PD-L1/PD-L2 + CTLA4 blocker indices did not markedly differ between the two groups. Subsequently, we explored anti-tumor drug sensitivity based on hub genes. Poor survival patients with downregulated SPRR1A and ALOX12B were sensitive to cisplatin, rapamycin, Idelalisib, everolimus (Fig. 8I), and were resistant to lapatinib, and afatinib, fluorouracil, and PHA-793887(Fig. S2, Fig. S3). We also observed that CSTA downregulation was an indicative of docetaxel resistance (Fig. S2, Fig. S3).

### Correlation between survival-related hub DEGs and immune/stromal cell infiltration determined in TIMER2.0

**Fig. 9:**
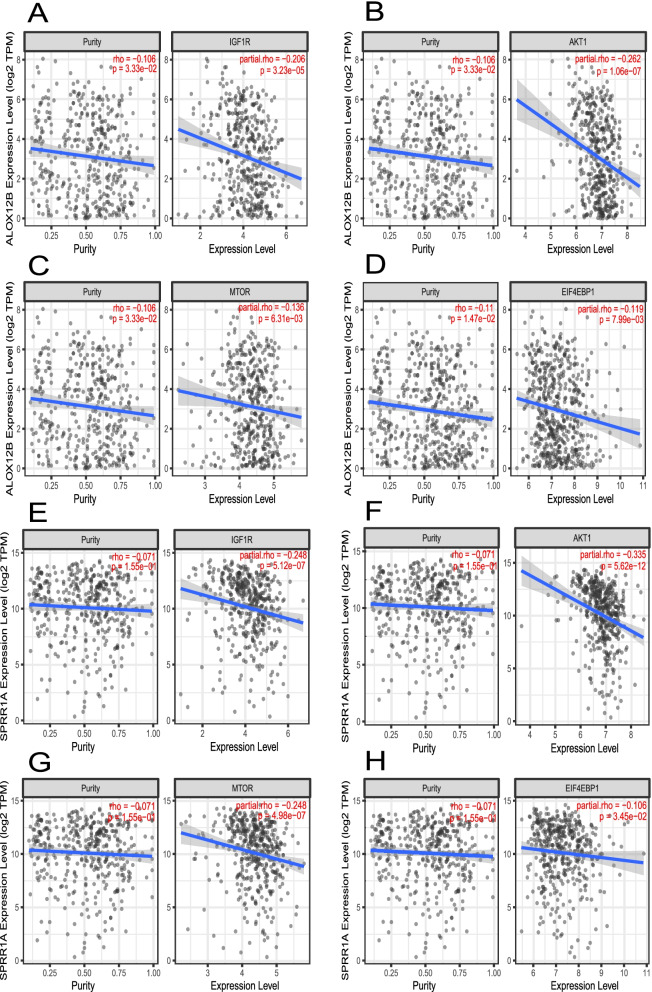
Association between the mRNA expression of ALOX12B (**A**-**D**) or SPRR1A (**E**-**H**) and the expression of insulin like growth factor 1 receptor (IGF1R), AKT serine/threonine kinase 1 (AKT1), mammalian target of rapamycin (MTOR), and eukaryotic translation initiation factor 4E binding protein 1 (EIF4EBP1) in HPV- HNSCC samples at TIMER2.0 online database

In the HPV- HNSCC TCGA database (TIMER2.0 online database), the xCell, EPIC, MCP-counter, and TIDE algorithms showed that downregulation of both hub genes (ALOX12B and SPRR1A) were correlated with increased cancer-associated fibroblasts (CAFs) (Table 4). The downstream effector of CAFs contained FAP, IGF1/2, PDGFs, IL6, TGFβ, LIF, NT5E, ADORA2B, CCL2/5, CXCL12, and CXCR4 [35, 36]. Table 5 shows that nearly all the foregoing effectors were negatively associated with the expression of ALOX12B and SPRR1A. After adjusting tumor purity, the expression of ALOX12B was negatively associated with IGFR1, AKT1, MTOR, and EIF4EBP1, and the same correlation could also be observed in SPRR1A (Fig. 9).

**Table 4:** The correlation analysis between the expression of SPRR1A and ALOX12B and the amount of infiltrated cancer associated fibroblasts

Gene	Immune cell	Spearman’s coefficient	***P*** value	Adjusted ***p*** value
**SPRR1A**	CAF_EPIC	−0.33	1.32E-11	4.50E-10
	CAF_MCP-counter	−0.37	1.55E-14	1.24E-12
	CAF_TIDE	−0.37	1.21E-14	1.24E-12
	CAF_XCELL	−0.26	1.53E-07	1.88E-06
**ALOX12B**	CAF_EPIC	−0.20	3.91E-05	0.0005
	CAF_MCP-counter	−0.25	3.71E-07	9.89E-06
	CAF_TIDE	−0.27	7.24E-08	2.32E-06
	CAF_XCELL	− 0.25	6.20E-07	1.42E-05

EPIC, MCP-counter, TIDE, and XCELL are algorithm for calculating the amount of immune cell infiltration in samples, *CAF* Cancer associated fibroblasts

**Table 5:** The correlation analysis between the expression of SPRR1A and ALOX12B and the expression of cancer associated fibroblasts effector

Down-effector gene	Hub gene	Spearman’s coefficient	***P*** value	Adjusted ***p*** value
ADORA2B	ALOX12B	−0.1703	0.0004	0.0013
ADORA2B	SPRR1A	−0.1822	0.0002	0.0011
CCL2	ALOX12B	−0.1225	0.0118	0.0882
CCL2	SPRR1A	−0.1893	0.0001	0.0014
CCL5	ALOX12B	−0.1719	0.0004	0.0022
CCL5	SPRR1A	−0.2548	< 0.001	< 0.001
CXCL12	ALOX12B	−0.2081	< 0.001	0.0003
CXCL12	SPRR1A	−0.2720	< 0.001	< 0.001
CXCR4	ALOX12B	−0.1739	0.0005	0.0024
CXCR4	SPRR1A	−0.2515	< 0.001	< 0.001
FAP	ALOX12B	−0.3224	< 0.001	< 0.001
FAP	SPRR1A	−0.4550	< 0.001	< 0.001
IGF1	ALOX12B	−0.1402	0.0050	0.0331
IGF1	SPRR1A	−0.1742	0.0005	0.0037
IGF2	ALOX12B	−0.0738	0.1405	0.3779
IGF2	SPRR1A	−0.1439	0.0039	0.0315
IL6	ALOX12B	−0.1210	0.0129	0.0573
IL6	SPRR1A	−0.1861	0.0002	0.0022
LIF	ALOX12B	−0.2408	< 0.001	< 0.001
LIF	SPRR1A	−0.2480	< 0.001	< 0.001
NT5E	ALOX12B	−0.2220	< 0.001	0.0002
NT5E	SPRR1A	−0.2635	< 0.001	< 0.001
PDGFA	ALOX12B	−0.3593	< 0.001	< 0.001
PDGFA	SPRR1A	−0.4350	< 0.001	< 0.001
PDGFB	ALOX12B	−0.2685	< 0.001	< 0.001
PDGFB	SPRR1A	−0.3193	< 0.001	< 0.001
PDGFC	ALOX12B	−0.2454	< 0.001	< 0.001
PDGFC	SPRR1A	−0.3551	< 0.001	< 0.001
PDGFD	ALOX12B	−0.1346	0.0070	0.0256
PDGFD	SPRR1A	−0.2002	0.0001	0.0003
TGFB1	ALOX12B	−0.1009	0.0436	0.1246
TGFB1	SPRR1A	−0.1852	0.0002	0.0027
TGFB2	ALOX12B	−0.2396	< 0.001	< 0.001
TGFB2	SPRR1A	−0.3247	< 0.001	< 0.001

### ALOX12B suppressed invasion and migration of HPV- HNSCC cell

**Fig. 10:**
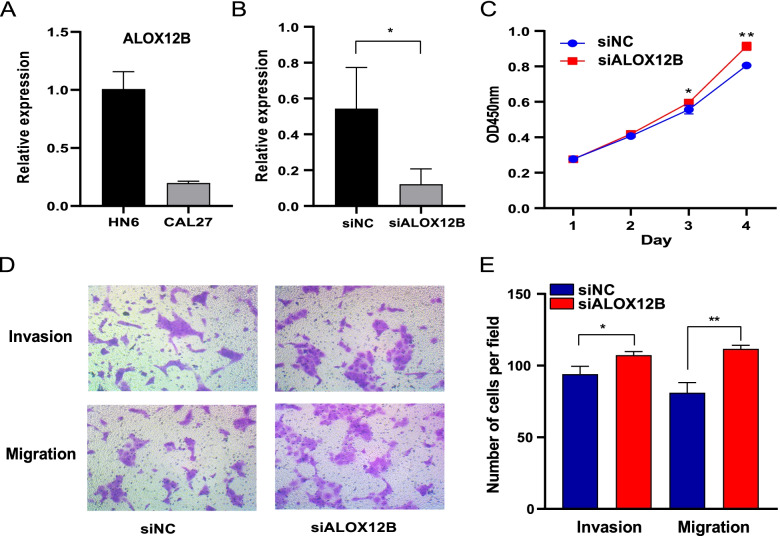
ALOX12B suppresses the migration and metastasis of HN6 cells in vitro. **A** Quantification mRNA expression of ALOX12B in HN6 and CAL27 cell lines by qRT-PCR. **B** HN6 cells were transfected with siNC and siALOX12B, and the expression of ALOX12B was examined using qRT-PCR. **C** CCK8 assay was conducted to assess cell viability in HN6-siNC and HN6-siALOX12B cells. **D**-**E** Transwell assays were performed to examine the invasion and migration of HN6-siNC and HN6-siALOX12B cells. Column, mean; Error bars, S.D.; *p < 0.05; **p < 0.01; ALOX12B, Arachidonate 12-Lipoxygenase, 12R Type; qRT-PCR, quantitative real-time polymerase chain reaction

The mRNA expression of ALOX12B was tested in two HPV- HNSCC cell lines, and the basal expression level of ALOX12B was high in HN6 cell line (Fig. 10A). Therefore, we knock down ALOX12B in HN6 cell line by siRNA, and the expression of ALOX12B was found to be suppressed using qRT-PCR (*P* < 0.05) (Fig. 10B). Knockdown of ALOX12B promoted HN6 cell proliferation in the CCK8 cell viability assay (*P* < 0.05) (Fig. 10C). Besides, transwell assays demonstrated that the knockdown of ALOX12B increased the migratory (*P* < 0.01) and invasive (*P* < 0.05) ability of HN6 cell (Fig. 10D-E).

## Discussion

Based on the transcriptional expression profiles of HPV16-HNSCC patients, we found that high DNA damage levels or low tumor microenvironment scores were associated with poor prognosis in HPV16- HNSCC patients. In addition, there were significant differences in the enriched core signaling pathways between the DDR_high/TM_high group and the DDR_high/TM_low group, and the DDR_high/TM_high group was enriched in immune cell development, polarization and activity-related signaling pathway, while the DDR_high/TM_low group was enriched in glutathione metabolism, drug metabolizing enzymes, platinum resistance, and ferroptosis. In multivariate survival analysis, we identified ALOX12B and SPRR1A as two protective survival-related genes in HPV16- HNSCC and found that the expression of the above two genes were negatively correlated with CAFs infiltration. We also showed the mRNA expression of ALOX12B and SPRR1A was negatively correlated with the mRNA expression of IGF1R, AKT1, MTOR, and EIF4EBP1 in HPV- HNSCC. In addition, downregulation the expression of ALOX12B promoted the invasion and migration ability of HPV- HNSCC cell.

WGCNA clusters genes with highly similar biological functions into a single module [37]. In the DDR_high/TM_low group, the positively correlated black and brown modules were enriched in several pathways. In the black module, the glycosphingolipid biosynthesis-lacto and neolacto series pathway was upregulated. A recent study revealed that an increased neolacto-series glycosphingolipid on the membrane of tumor cells hinders the interaction between HLA-I and CD8+ T cells, and then impedes CD8 + T cell activation [38]. In the brown module, glutathione metabolism and drug metabolism-cytochrome P450 pathways were enriched. Previously studies reported that cancer stem cells can scavenge intracellular reactive oxygen species (ROS) through glutathione or resistant to treatment through abnormal drug metabolizing enzyme pathways [39–41]. We also noticed the cancer stem cell gene signature SOX2 and EPCAM was clustered in the brown module, moreover, cancer stem cell has been reported exhibiting resistance to immunotherapy [42–44]. Besides, ferroptosis pathway that enriched in brown module was disturbed during epithelial-to-mesenchymal transition (EMT) process [45]. In the DDR_high/TM_high group, the positively correlated blue module was enriched the neutrophil extracellular traps (NETs) pathway and PD − L1 expression and PD − 1 checkpoint pathway in cancer. We note that a study has shown that the barrier formed by NETs impedes the cell-to-cell contact between tumor cells and CD8+ T cells, and then hinders the antitumor activity of cytotoxic T cells, and simultaneously targeting PD-1 and NETs can increase tumor regression in vivo [46]. The mutation of a single gene can reflect the immune status to a certain degree. An example is the MUC16 mutation status for gastric cancer [47]. Our somatic mutation analysis showed an increased NSD1 mutation frequency in the DDR_high/TM_low group and a high CASP8 mutation rate in the DDR_high/TM_high group. The results were consistent with previous reports that NSD1 mutation is an intrinsic feature of cold immune phenotype, and the frequency of CASP8 mutation is increased in HNSCC with hot immune phenotype [48, 49].

SPRR1A and ALOX12B downregulation was observed in HPV16- HNSCC patients at advanced cancer stage. SPRR1A expression was positively associated with favorable survival and lower lymph node metastasis in HNSCC patients [50]. ALOXE3 as a paralog of ALOX12B, inhibits glioblastoma tumor migration [51]. Our results revealed that ALOX12B is an independent protective prognostic indicator in HPV16- HNSCC patients. However, another study revealed that ALOX12B mediates cervical cancer cell proliferation and migration via the PI3K-ERK1 pathway [52]. We also noticed that both hub genes (SPRR1A and ALOX12B) participate in epidermal cell differentiation and the skin barrier. ALOX12B participates in constructing the mature corneocyte lipid envelope [53]. SPRR1A is a structural component of the epidermis [54]. Mutation of ALOX12B and/or SPRR1A may result in skin barrier-related diseases, such as psoriasis and autosomal-recessive exfoliative ichthyosis [55]. ZNF750 and GLIS1 that regulating ALOX12B and SPRR1A expression belong to Zinc-finger proteins (ZNFs) [55]. ZNF750 is recognized as a tumor suppressor gene in HNSCC, and ALOX12B downregulation may indirectly reflect a loss of ZNFs expression in advanced HNSCC [56, 57]. A single-cell transcriptomic research including HPV16- HNSCC samples assessed the gene signatures of epithelial differentiation. SPRR1A and ZNF750 were listed in the top 50 genes of epithelial differentiation characteristic genes [58]. A low epithelial differentiation score was negatively correlated with a high partial epithelial-to-mesenchymal transition (p-EMT) score in the mesenchymal and basal subtype. The proportion of mesenchymal and basal subtypes increased with the elevating of p-EMT score. Patients classified as mesenchymal and basal subtypes showed poor survival in the whole TCGA cohort. Hence, a low epithelial score could serve as a surrogate for worse survival in HPV16- HNSCC patients.

The xCell and MCP-counter algorithms used the gene marker-based method while EPIC was based on the deconvolution approach [59]. All four algorithms showed that the down-regulation of ALOX12B and SPRR1A genes was correlated with increased CAFs infiltration in HPV16- HNSCC. A study demonstrated SPRR1A downregulation in MCF-10A breast cancer cells when coculture with CAFs [60]. CAFs promote tumor progression via promoting EMT and metabolic reprogramming [61]. These vicious behaviors are achieved via the expression of specific membrane proteins and paracrine cytokines or chemokines, such as FAP, IL6, TGFβ, LIF, NT5E, ADORA2B, CCL2/5, CXCL12, CXCR4, IL7, IGF1/2 [35, 36]. Our research indicated that the ALOX12B and SPRR1A expression levels were nearly always negatively correlated with the above effectors. Thus, both genes potentially reflect infiltrated CAFs quantity and quality. However, another study identified ALOX12B as an immunosuppressive factor based on a cytolytic activity analysis [49]. Therefore, the integrated roles of ALOX12B in the tumor microenvironment (CD8+ T cell, CAFs, regulatory T cells, myeloid-derived suppressor cells and so on) merit further investigation.

A docetaxel plus cisplatin and fluorouracil (TPF) chemotherapy regimen is recommended as the induction therapy for patients with stage III-IV in head and neck cancer [62]. In the present study, we observed that the downregulation of SPRR1A was associated with high sensitivity of cisplatin, and upregulation of ALOX12B and CSTA was associated with high sensitivity of fluorouracil, and docetaxel, respectively. LUX-Head & Neck 1 trial showed the efficacy of afatinib as a second-line treatment for recurrence or metastatic (RM) HNSCC patients [63]. We found that SPRR1A and CSTA expression were correlated with afatinib sensitivity. A phase II trial reported the efficiency of lapatinib and capecitabine therapy against RM HNSCC [64], and our results showed an association between SPRR1A and CSTA expression with lapatinib response. We also found that high ALOX12B expression was sensitive to CTLA4 inhibitors by calculating IPS-CTLA4 blocker scores. Notably, our research showed the expression of ALOX12B was negatively correlated with CAFs infiltration, and decreased ALOX12B expression indicated a better response to rapamycin or everolimus (drug target for mTOR). We try to explain the result in this way: The proliferation and development of CAFs are regulated by PI3K/AKT/mTOR signaling pathway, and CAFs might promote tumor progress via IGF1R/AKT1/mTOR pathway in tumor microenvironment [65, 66]. Moreover, for patients with head and neck squamous cell carcinoma, the level of phosphorylated mTOR in the junction zone between tumor and normal tissue or in tumor area was higher than that in the normal mucosal tissue, and the level of phosphorylated mTOR in the junction zone was higher than that in tumor area [67]. Meanwhile, upregulated mTOR expression predicted poor overall survival in HPV16- HNSCC patients [68]. The combination of everolimus plus docetaxel represented greater tumor regression than the use of docetaxel alone in a nude mouse xenograft model [69]. Therefore, poor prognosis patients with low ALOX12B expression had high infiltration of CAFs surrounding tumor cells, and rapamycin or everolimus may provide survival benefits by inhibiting mTOR signaling.

Our study has several limitations: 1, The clinical value of ALOX12B and SPRR1A was only validated in one GEO data set. Considering the different anatomical sites in HNSCC have different transcriptome profiles [70], TCGA and GSE65858 data set contained mixed HNSCC samples, while the GSE41613 data set only included oral squamous cell carcinoma samples. Thus, the difference in the anatomy of the two external verification data sets may be the reason for the different verification results, and further prospective research is required to verify the prognostic and drug sensitivity value of ALOX12B and SPRR1A in HPV16- HNSCC; 2, The specific mechanism of the abnormal expression of ALOX12B affecting the invasion and metastasis of HPV-HNSCC cell needs to be revealed by further experiments in vitro and in vivo; 3, The association between hub genes and drug sensitivity in CellMiner platform was based on NCI-60 tumor cells, while the cell assay lacks HNSCC cells. Besides, a zero-inflation data was used to analyse the correlation of the expression of SPRR1A and cisplatin sensitivity, and the reliability of this association needs to be further verified. Thus, the identified drug pattern based on hub genes needs to be validated in a patient-derived xenograft model.

## Conclusion

Our bioinformatics analysis indicated that the intrinsic DNA repair level and tumor microenvironment status were associated with prognosis in HPV16- HNSCC patients. We identified two hub genes ALOX12B and SPRR1A, and showed that they can predict the clinical outcomes of HPV16- HNSCC. In addition, the two genes may be indicators of the amount of infiltrated CAFs. Nevertheless, further clinical research is required to validate drug sensitivity based on the expression of the those genes.

## Supplementary Information

**Additional file 1.**

## Data Availability

The data presented in this study are included in the article/Supplementary Material, further inquiries are available from the corresponding author on reasonable request.

## Abbreviations

AKT1: AKT serine/threonine kinase 1
BER: Base excision repair
CAFs: Cancer-associated fibroblasts
CLE: Corneocyte lipid envelope
CTRP: Cancer Therapeutics Response Portal
DDR: DNA damage response
DEG: Differentially expressed gene
EGFR: Epidermal growth factor receptor
EIF4EBP1: Eukaryotic translation initiation factor 4E binding protein 1
EMT: Epithelial-to-mesenchymal transition
FDR: False discovery rate
FPKM: Fragments per kilobase per million reads
GDSC: Genomics of Drug Sensitivity in Cancer
GEO: Gene Expression Omnibus
HNSCC: Head and neck squamous cell carcinoma
HPV: Human papillomavirus
HRR: Homologous recombination repair
IGF: Insulin-like growth factor
IGF1R: Insulin like growth factor 1 receptor
IPS: Immunophenoscore
KEGG: Kyoto Encyclopedia of Genes and Genomes
KM: Kaplan-Meier
MHC: Major histocompatibility complex
MMR: Mismatch repair
MSigDB: Molecular Signatures database
MTOR: Mammalian target of rapamycin
NER: Nucleotide excision repair
NETs: Neutrophil extracellular traps
NHEJ: Nonhomologous end joining
OS: Overall survival
p-EMT: Partial epithelial-to-mesenchymal transition
PPI: Protein-protein interaction
RM: Recurrence/metastasis
TCGA: The Cancer Genome Atlas
TCIA: The Cancer Immunome Atlas
TM: Tumor microenvironment
TNM: Tumor, node and metastasis
TOM: Topology overlap matrix
WGCNA: Weighted gene co-expression network analysis
ZNF: Zinc finger protein
