# Correction: Survival-related indicators ALOX12B and SPRR1A are associated with DNA damage repair and tumor microenvironment status in HPV 16-negative head and neck squamous cell carcinoma patients

**DOI:** 10.1186/s12885-022-09865-x

**Published:** 2022-07-11

**Authors:** Jing Li, Ling-Long Tang, Jun Ma

**Affiliations:** 1 Department of Radiation Oncology, Sun Yat-sen University Cancer Center, 651 Dongfeng Road East, Guangzhou, 510060 Guangdong China; 2 State Key Laboratory of Oncology in South China, Collaborative Innovation Center for Cancer Medicine, 651 Dong feng Road East, Guangzhou, 510060 China

**Correction: BMC Cancer 22, 714 (2022)**

**https://doi.org/10.1186/s12885-022-09722-x**

Following publication of the original article [1], the authors identified an error in the results of abstract section.

The description in the results of abstract section in this article currently reads “… while the DDR_high/TM_low samples were enriched in Th17 cell diferentiation, ...”. This is incorrect and should read as follows “… while the DDR_high/TM_high samples were enriched in Th17 cell diferentiation, ....”.
