# Supplementary material for: Survival-related indicators ALOX12B and SPRR1A are associated with DNA damage repair and tumor microenvironment status in HPV 16-negative head and neck squamous cell carcinoma patients
Source: BMC Cancer. 2022 Jun 29;22:714. doi: 10.1186/s12885-022-09722-x (PMC9241267; doi:10.1186/s12885-022-09722-x)

**Supplementary Table S1.** Identity document (ID) and human papillomavirus infection status of patients included in the TCGA discovery cohort and GSE65858 data set

| TCGA.PATIENT.ID | HPV |
|---|---|
| TCGA-4P-AA8J-01 | NA |
| TCGA-BA-4074-01 | NA |
| TCGA-BA-4075-01 | NA |
| TCGA-BA-4076-01 | NA |
| TCGA-BA-4078-01 | NA |
| TCGA-BA-5151-01 | NA |
| TCGA-BA-5555-01 | NA |
| TCGA-BA-5556-01 | NA |
| TCGA-BA-5557-01 | NA |
| TCGA-BA-5558-01 | NA |
| TCGA-BA-6868-01 | NA |
| TCGA-BA-6869-01 | NA |
| TCGA-BA-6870-01 | NA |
| TCGA-BA-6871-01 | NA |
| TCGA-BA-6872-01 | NA |
| TCGA-BA-6873-01 | NA |
| TCGA-BA-7269-01 | NA |
| TCGA-BA-A4IF-01 | NA |
| TCGA-BA-A6D8-01 | NA |
| TCGA-BA-A6DA-01 | NA |
| TCGA-BA-A6DB-01 | NA |
| TCGA-BA-A6DD-01 | NA |
| TCGA-BA-A6DE-01 | NA |
| TCGA-BA-A6DG-01 | NA |
| TCGA-BA-A6DI-01 | NA |
| TCGA-BA-A6DJ-01 | NA |
| TCGA-BA-A6DL-01 | NA |
| TCGA-BA-A8YP-01 | NA |
| TCGA-BB-4217-01 | NA |
| TCGA-BB-4225-01 | hpv33 |
| TCGA-BB-4227-01 | hpv33 |
| TCGA-BB-8596-01 | NA |
| TCGA-BB-8601-01 | NA |
| TCGA-BB-A5HU-01 | NA |
| TCGA-BB-A5HY-01 | NA |
| TCGA-BB-A5HZ-01 | NA |
| TCGA-C9-A47Z-01 | NA |
| TCGA-C9-A480-01 | NA |
| TCGA-CN-4722-01 | NA |
| TCGA-CN-4723-01 | NA |
| TCGA-CN-4725-01 | NA |

| | |
|---|---|
| TCGA-CN-4726-01 | NA |
| TCGA-CN-4727-01 | NA |
| TCGA-CN-4728-01 | NA |
| TCGA-CN-4729-01 | NA |
| TCGA-CN-4730-01 | NA |
| TCGA-CN-4731-01 | NA |
| TCGA-CN-4733-01 | NA |
| TCGA-CN-4734-01 | NA |
| TCGA-CN-4735-01 | NA |
| TCGA-CN-4736-01 | NA |
| TCGA-CN-4737-01 | NA |
| TCGA-CN-4738-01 | NA |
| TCGA-CN-4739-01 | NA |
| TCGA-CN-4740-01 | NA |
| TCGA-CN-4742-01 | NA |
| TCGA-CN-5355-01 | NA |
| TCGA-CN-5356-01 | NA |
| TCGA-CN-5358-01 | NA |
| TCGA-CN-5359-01 | NA |
| TCGA-CN-5360-01 | NA |
| TCGA-CN-5361-01 | NA |
| TCGA-CN-5363-01 | NA |
| TCGA-CN-5367-01 | NA |
| TCGA-CN-5369-01 | NA |
| TCGA-CN-5370-01 | NA |
| TCGA-CN-5373-01 | NA |
| TCGA-CN-6010-01 | NA |
| TCGA-CN-6011-01 | NA |
| TCGA-CN-6012-01 | NA |
| TCGA-CN-6013-01 | NA |
| TCGA-CN-6016-01 | NA |
| TCGA-CN-6017-01 | NA |
| TCGA-CN-6018-01 | NA |
| TCGA-CN-6019-01 | NA |
| TCGA-CN-6020-01 | NA |
| TCGA-CN-6021-01 | NA |
| TCGA-CN-6022-01 | NA |
| TCGA-CN-6023-01 | NA |
| TCGA-CN-6024-01 | NA |
| TCGA-CN-6988-01 | NA |
| TCGA-CN-6989-01 | NA |
| TCGA-CN-6992-01 | NA |
| TCGA-CN-6994-01 | NA |
| TCGA-CN-6995-01 | NA |

| | |
|---|---|
| TCGA-CN-6996-01 | NA |
| TCGA-CN-6997-01 | NA |
| TCGA-CN-6998-01 | NA |
| TCGA-CN-A497-01 | NA |
| TCGA-CN-A498-01 | NA |
| TCGA-CN-A49A-01 | NA |
| TCGA-CN-A49B-01 | NA |
| TCGA-CN-A63T-01 | NA |
| TCGA-CN-A63U-01 | NA |
| TCGA-CN-A63V-01 | NA |
| TCGA-CN-A63W-01 | NA |
| TCGA-CN-A641-01 | NA |
| TCGA-CN-A642-01 | NA |
| TCGA-CN-A6V3-01 | NA |
| TCGA-CQ-5324-01 | NA |
| TCGA-CQ-5325-01 | NA |
| TCGA-CQ-5326-01 | NA |
| TCGA-CQ-5327-01 | NA |
| TCGA-CQ-5329-01 | NA |
| TCGA-CQ-5330-01 | NA |
| TCGA-CQ-5331-01 | NA |
| TCGA-CQ-5332-01 | NA |
| TCGA-CQ-5333-01 | NA |
| TCGA-CQ-5334-01 | NA |
| TCGA-CQ-6218-01 | NA |
| TCGA-CQ-6219-01 | NA |
| TCGA-CQ-6220-01 | NA |
| TCGA-CQ-6221-01 | NA |
| TCGA-CQ-6222-01 | NA |
| TCGA-CQ-6223-01 | NA |
| TCGA-CQ-6224-01 | NA |
| TCGA-CQ-6225-01 | NA |
| TCGA-CQ-6227-01 | NA |
| TCGA-CQ-6228-01 | NA |
| TCGA-CQ-6229-01 | NA |
| TCGA-CQ-7063-01 | NA |
| TCGA-CQ-7065-01 | NA |
| TCGA-CQ-7068-01 | NA |
| TCGA-CQ-7069-01 | NA |
| TCGA-CQ-7071-01 | NA |
| TCGA-CQ-7072-01 | NA |
| TCGA-CQ-A4C6-01 | NA |
| TCGA-CQ-A4C7-01 | NA |
| TCGA-CQ-A4C9-01 | NA |

| | |
|---|---|
| TCGA-CQ-A4CA-01 | NA |
| TCGA-CQ-A4CB-01 | NA |
| TCGA-CQ-A4CD-01 | NA |
| TCGA-CQ-A4CE-01 | NA |
| TCGA-CQ-A4CG-01 | NA |
| TCGA-CQ-A4CH-01 | NA |
| TCGA-CQ-A4CI-01 | NA |
| TCGA-CR-5247-01 | NA |
| TCGA-CR-6467-01 | hpv33 |
| TCGA-CR-6471-01 | hpv33 |
| TCGA-CR-6477-01 | NA |
| TCGA-CR-6478-01 | NA |
| TCGA-CR-6488-01 | NA |
| TCGA-CR-6492-01 | NA |
| TCGA-CR-7364-01 | NA |
| TCGA-CR-7365-01 | NA |
| TCGA-CR-7367-01 | NA |
| TCGA-CR-7370-01 | NA |
| TCGA-CR-7371-01 | NA |
| TCGA-CR-7372-01 | NA |
| TCGA-CR-7373-01 | NA |
| TCGA-CR-7374-01 | NA |
| TCGA-CR-7376-01 | NA |
| TCGA-CR-7377-01 | NA |
| TCGA-CR-7379-01 | hpv18 |
| TCGA-CR-7380-01 | NA |
| TCGA-CR-7382-01 | NA |
| TCGA-CR-7383-01 | NA |
| TCGA-CR-7386-01 | NA |
| TCGA-CR-7388-01 | NA |
| TCGA-CR-7389-01 | NA |
| TCGA-CR-7390-01 | NA |
| TCGA-CR-7391-01 | NA |
| TCGA-CR-7392-01 | NA |
| TCGA-CR-7393-01 | NA |
| TCGA-CR-7394-01 | NA |
| TCGA-CR-7395-01 | NA |
| TCGA-CR-7397-01 | NA |
| TCGA-CR-7398-01 | NA |
| TCGA-CR-7399-01 | NA |
| TCGA-CR-7401-01 | hpv18 |
| TCGA-CR-7402-01 | hpv18 |
| TCGA-CR-7404-01 | NA |
| TCGA-CV-5430-01 | NA |

| | |
|---|---|
| TCGA-CV-5431-01 | NA |
| TCGA-CV-5432-01 | NA |
| TCGA-CV-5434-01 | NA |
| TCGA-CV-5435-01 | NA |
| TCGA-CV-5436-01 | NA |
| TCGA-CV-5439-01 | NA |
| TCGA-CV-5440-01 | NA |
| TCGA-CV-5441-01 | NA |
| TCGA-CV-5444-01 | NA |
| TCGA-CV-5966-01 | NA |
| TCGA-CV-5970-01 | NA |
| TCGA-CV-5973-01 | NA |
| TCGA-CV-5976-01 | NA |
| TCGA-CV-5977-01 | NA |
| TCGA-CV-5978-01 | NA |
| TCGA-CV-5979-01 | NA |
| TCGA-CV-6003-01 | NA |
| TCGA-CV-6436-01 | NA |
| TCGA-CV-6441-01 | NA |
| TCGA-CV-6933-01 | NA |
| TCGA-CV-6934-01 | NA |
| TCGA-CV-6935-01 | NA |
| TCGA-CV-6936-01 | NA |
| TCGA-CV-6937-01 | NA |
| TCGA-CV-6938-01 | NA |
| TCGA-CV-6939-01 | hpv33 |
| TCGA-CV-6940-01 | NA |
| TCGA-CV-6941-01 | hpv33 |
| TCGA-CV-6942-01 | NA |
| TCGA-CV-6943-01 | NA |
| TCGA-CV-6945-01 | NA |
| TCGA-CV-6948-01 | NA |
| TCGA-CV-6950-01 | NA |
| TCGA-CV-6951-01 | NA |
| TCGA-CV-6952-01 | NA |
| TCGA-CV-6953-01 | NA |
| TCGA-CV-6954-01 | NA |
| TCGA-CV-6955-01 | NA |
| TCGA-CV-6956-01 | NA |
| TCGA-CV-6959-01 | NA |
| TCGA-CV-6960-01 | NA |
| TCGA-CV-6962-01 | NA |
| TCGA-CV-7089-01 | NA |
| TCGA-CV-7090-01 | NA |

| | |
|---|---|
| TCGA-CV-7091-01 | NA |
| TCGA-CV-7095-01 | NA |
| TCGA-CV-7097-01 | hpv33 |
| TCGA-CV-7099-01 | NA |
| TCGA-CV-7100-01 | hpv33 |
| TCGA-CV-7101-01 | hpv33 |
| TCGA-CV-7102-01 | hpv33 |
| TCGA-CV-7103-01 | hpv33 |
| TCGA-CV-7104-01 | hpv33 |
| TCGA-CV-7177-01 | NA |
| TCGA-CV-7178-01 | NA |
| TCGA-CV-7180-01 | NA |
| TCGA-CV-7183-01 | NA |
| TCGA-CV-7235-01 | NA |
| TCGA-CV-7236-01 | NA |
| TCGA-CV-7238-01 | NA |
| TCGA-CV-7242-01 | NA |
| TCGA-CV-7245-01 | NA |
| TCGA-CV-7247-01 | NA |
| TCGA-CV-7248-01 | NA |
| TCGA-CV-7250-01 | NA |
| TCGA-CV-7252-01 | NA |
| TCGA-CV-7253-01 | NA |
| TCGA-CV-7254-01 | NA |
| TCGA-CV-7255-01 | NA |
| TCGA-CV-7261-01 | NA |
| TCGA-CV-7263-01 | NA |
| TCGA-CV-7407-01 | NA |
| TCGA-CV-7410-01 | NA |
| TCGA-CV-7411-01 | NA |
| TCGA-CV-7413-01 | NA |
| TCGA-CV-7414-01 | NA |
| TCGA-CV-7415-01 | NA |
| TCGA-CV-7416-01 | NA |
| TCGA-CV-7418-01 | NA |
| TCGA-CV-7421-01 | NA |
| TCGA-CV-7422-01 | NA |
| TCGA-CV-7423-01 | NA |
| TCGA-CV-7424-01 | NA |
| TCGA-CV-7425-01 | NA |
| TCGA-CV-7427-01 | NA |
| TCGA-CV-7428-01 | NA |
| TCGA-CV-7429-01 | NA |
| TCGA-CV-7430-01 | NA |

| | |
|---|---|
| TCGA-CV-7432-01 | NA |
| TCGA-CV-7433-01 | NA |
| TCGA-CV-7434-01 | NA |
| TCGA-CV-7435-01 | NA |
| TCGA-CV-7437-01 | NA |
| TCGA-CV-7438-01 | NA |
| TCGA-CV-7440-01 | NA |
| TCGA-CV-7446-01 | NA |
| TCGA-CV-7568-01 | NA |
| TCGA-CV-A45O-01 | NA |
| TCGA-CV-A45P-01 | NA |
| TCGA-CV-A45Q-01 | NA |
| TCGA-CV-A45R-01 | NA |
| TCGA-CV-A45T-01 | NA |
| TCGA-CV-A45U-01 | NA |
| TCGA-CV-A45V-01 | NA |
| TCGA-CV-A45W-01 | NA |
| TCGA-CV-A45X-01 | NA |
| TCGA-CV-A45Y-01 | NA |
| TCGA-CV-A45Z-01 | NA |
| TCGA-CV-A460-01 | NA |
| TCGA-CV-A461-01 | NA |
| TCGA-CV-A463-01 | NA |
| TCGA-CV-A464-01 | NA |
| TCGA-CV-A465-01 | NA |
| TCGA-CV-A468-01 | NA |
| TCGA-CV-A6JD-01 | NA |
| TCGA-CV-A6JE-01 | NA |
| TCGA-CV-A6JM-01 | NA |
| TCGA-CV-A6JN-01 | NA |
| TCGA-CV-A6JO-01 | NA |
| TCGA-CV-A6JT-01 | NA |
| TCGA-CV-A6JU-01 | NA |
| TCGA-CV-A6JY-01 | NA |
| TCGA-CV-A6JZ-01 | NA |
| TCGA-CV-A6K0-01 | NA |
| TCGA-CV-A6K1-01 | NA |
| TCGA-CV-A6K2-01 | NA |
| TCGA-CX-7082-01 | NA |
| TCGA-CX-7085-01 | NA |
| TCGA-CX-7086-01 | NA |
| TCGA-CX-7219-01 | NA |
| TCGA-CX-A4AQ-01 | NA |
| TCGA-D6-6515-01 | NA |

| | |
|---|---|
| TCGA-D6-6516-01 | NA |
| TCGA-D6-6517-01 | NA |
| TCGA-D6-6823-01 | NA |
| TCGA-D6-6824-01 | NA |
| TCGA-D6-6825-01 | NA |
| TCGA-D6-6826-01 | NA |
| TCGA-D6-6827-01 | NA |
| TCGA-D6-8568-01 | NA |
| TCGA-D6-8569-01 | NA |
| TCGA-D6-A4Z9-01 | NA |
| TCGA-D6-A4ZB-01 | NA |
| TCGA-D6-A6EK-01 | NA |
| TCGA-D6-A6EM-01 | NA |
| TCGA-D6-A6EN-01 | NA |
| TCGA-D6-A6EO-01 | NA |
| TCGA-D6-A6EQ-01 | NA |
| TCGA-D6-A6ES-01 | NA |
| TCGA-D6-A74Q-01 | NA |
| TCGA-DQ-5624-01 | NA |
| TCGA-DQ-5625-01 | NA |
| TCGA-DQ-5630-01 | NA |
| TCGA-DQ-7588-01 | NA |
| TCGA-DQ-7591-01 | hpv35 |
| TCGA-DQ-7592-01 | NA |
| TCGA-F7-7848-01 | NA |
| TCGA-F7-8298-01 | NA |
| TCGA-F7-8489-01 | NA |
| TCGA-F7-A50G-01 | NA |
| TCGA-F7-A50I-01 | NA |
| TCGA-F7-A50J-01 | NA |
| TCGA-F7-A61S-01 | NA |
| TCGA-F7-A61V-01 | NA |
| TCGA-F7-A61W-01 | NA |
| TCGA-F7-A620-01 | NA |
| TCGA-F7-A622-01 | NA |
| TCGA-F7-A623-01 | NA |
| TCGA-F7-A624-01 | NA |
| TCGA-H7-7774-01 | NA |
| TCGA-H7-8501-01 | NA |
| TCGA-H7-8502-01 | NA |
| TCGA-H7-A6C4-01 | NA |
| TCGA-HD-7229-01 | NA |
| TCGA-HD-7753-01 | NA |
| TCGA-HD-7832-01 | hpv33 |

| | |
|---|---|
| TCGA-HD-8224-01 | NA |
| TCGA-HD-8314-01 | hpv35 |
| TCGA-HD-8634-01 | NA |
| TCGA-HD-8635-01 | NA |
| TCGA-HD-A4C1-01 | NA |
| TCGA-HD-A633-01 | NA |
| TCGA-HD-A6HZ-01 | NA |
| TCGA-HD-A6I0-01 | NA |
| TCGA-IQ-7630-01 | NA |
| TCGA-IQ-7631-01 | NA |
| TCGA-IQ-7632-01 | NA |
| TCGA-IQ-A61E-01 | NA |
| TCGA-IQ-A61G-01 | NA |
| TCGA-IQ-A61H-01 | NA |
| TCGA-IQ-A61I-01 | hpv35 |
| TCGA-IQ-A61J-01 | NA |
| TCGA-IQ-A61O-01 | NA |
| TCGA-IQ-A6SG-01 | NA |
| TCGA-IQ-A6SH-01 | NA |
| TCGA-KU-A66S-01 | NA |
| TCGA-KU-A66T-01 | NA |
| TCGA-KU-A6H8-01 | NA |
| TCGA-MT-A51W-01 | NA |
| TCGA-MT-A51X-01 | NA |
| TCGA-MT-A67A-01 | NA |
| TCGA-MT-A67D-01 | NA |
| TCGA-MT-A67F-01 | NA |
| TCGA-MT-A7BN-01 | NA |
| TCGA-MZ-A5BI-01 | hpv33 |
| TCGA-MZ-A7D7-01 | NA |
| TCGA-P3-A5Q6-01 | NA |
| TCGA-P3-A5QA-01 | NA |
| TCGA-P3-A6SX-01 | NA |
| TCGA-P3-A6T0-01 | NA |
| TCGA-P3-A6T2-01 | NA |
| TCGA-P3-A6T3-01 | NA |
| TCGA-P3-A6T4-01 | NA |
| TCGA-P3-A6T5-01 | NA |
| TCGA-P3-A6T7-01 | NA |
| TCGA-P3-A6T8-01 | NA |
| TCGA-QK-A64Z-01 | NA |
| TCGA-QK-A652-01 | NA |
| TCGA-QK-A6IG-01 | NA |
| TCGA-QK-A6IH-01 | NA |

| | |
|---|---|
| TCGA-QK-A6II-01 | NA |
| TCGA-QK-A6IJ-01 | NA |
| TCGA-QK-A6VB-01 | NA |
| TCGA-QK-A6VC-01 | NA |
| TCGA-QK-A8Z7-01 | NA |
| TCGA-QK-A8Z8-01 | NA |
| TCGA-QK-A8Z9-01 | NA |
| TCGA-QK-A8ZA-01 | NA |
| TCGA-QK-A8ZB-01 | NA |
| TCGA-QK-AA3J-01 | NA |
| TCGA-QK-AA3K-01 | NA |
| TCGA-RS-A6TO-01 | NA |
| TCGA-T2-A6WX-01 | NA |
| TCGA-T2-A6WZ-01 | NA |
| TCGA-T2-A6X2-01 | NA |
| TCGA-T3-A92M-01 | NA |
| TCGA-T3-A92N-01 | NA |
| TCGA-TN-A7HI-01 | hpv33 |
| TCGA-TN-A7HJ-01 | NA |
| TCGA-UF-A718-01 | NA |
| TCGA-UF-A719-01 | NA |
| TCGA-UF-A71A-01 | NA |
| TCGA-UF-A71A-06 | NA |
| TCGA-UF-A71B-01 | NA |
| TCGA-UF-A71D-01 | NA |
| TCGA-UF-A71E-01 | NA |
| TCGA-UF-A7J9-01 | NA |
| TCGA-UF-A7JA-01 | NA |
| TCGA-UF-A7JC-01 | NA |
| TCGA-UF-A7JD-01 | NA |
| TCGA-UF-A7JF-01 | NA |
| TCGA-UF-A7JH-01 | NA |
| TCGA-UF-A7JJ-01 | NA |
| TCGA-UF-A7JK-01 | NA |
| TCGA-UF-A7JO-01 | NA |
| TCGA-UF-A7JS-01 | NA |
| TCGA-UF-A7JT-01 | NA |
| TCGA-UF-A7JV-01 | NA |
| TCGA-WA-A7GZ-01 | NA |
| TCGA-WA-A7H4-01 | NA |
| **GSE65858_!Sample_geo_accession** | **GSE65858_!Sample_characteristics_ch1** |
| GSM1607689 | hpv_dna: Other HPV |
| GSM1607690 | hpv_dna: Other HPV |
| GSM1607691 | hpv_dna: Other HPV |

| | |
|---|---|
| GSM1607692 | hpv_dna: Negative |
| GSM1607693 | hpv_dna: Negative |
| GSM1607694 | hpv_dna: Negative |
| GSM1607695 | hpv_dna: Negative |
| GSM1607696 | hpv_dna: Negative |
| GSM1607698 | hpv_dna: Negative |
| GSM1607699 | hpv_dna: Negative |
| GSM1607700 | hpv_dna: Other HPV |
| GSM1607703 | hpv_dna: Other HPV |
| GSM1607723 | hpv_dna: Other HPV |
| GSM1607724 | hpv_dna: Other HPV |
| GSM1607725 | hpv_dna: Other HPV |
| GSM1607726 | hpv_dna: Negative |
| GSM1607727 | hpv_dna: Negative |
| GSM1607728 | hpv_dna: Negative |
| GSM1607729 | hpv_dna: Negative |
| GSM1607730 | hpv_dna: Negative |
| GSM1607731 | hpv_dna: Negative |
| GSM1607732 | hpv_dna: Negative |
| GSM1607733 | hpv_dna: Negative |
| GSM1607734 | hpv_dna: Negative |
| GSM1607735 | hpv_dna: Negative |
| GSM1607736 | hpv_dna: Negative |
| GSM1607737 | hpv_dna: Negative |
| GSM1607738 | hpv_dna: Negative |
| GSM1607739 | hpv_dna: Negative |
| GSM1607740 | hpv_dna: Negative |
| GSM1607741 | hpv_dna: Negative |
| GSM1607742 | hpv_dna: Negative |
| GSM1607743 | hpv_dna: Negative |
| GSM1607744 | hpv_dna: Negative |
| GSM1607745 | hpv_dna: Negative |
| GSM1607746 | hpv_dna: Negative |
| GSM1607747 | hpv_dna: Negative |
| GSM1607748 | hpv_dna: Negative |
| GSM1607749 | hpv_dna: Negative |
| GSM1607750 | hpv_dna: Negative |
| GSM1607751 | hpv_dna: Negative |
| GSM1607752 | hpv_dna: Negative |
| GSM1607753 | hpv_dna: Negative |
| GSM1607754 | hpv_dna: Negative |
| GSM1607755 | hpv_dna: Negative |
| GSM1607756 | hpv_dna: Negative |
| GSM1607757 | hpv_dna: Negative |

| | |
|---|---|
| GSM1607758 | hpv_dna: Negative |
| GSM1607759 | hpv_dna: Negative |
| GSM1607760 | hpv_dna: Negative |
| GSM1607761 | hpv_dna: Negative |
| GSM1607762 | hpv_dna: Negative |
| GSM1607763 | hpv_dna: Negative |
| GSM1607764 | hpv_dna: Negative |
| GSM1607766 | hpv_dna: NA |
| GSM1607767 | hpv_dna: Negative |
| GSM1607768 | hpv_dna: Negative |
| GSM1607769 | hpv_dna: Negative |
| GSM1607770 | hpv_dna: Other HPV |
| GSM1607773 | hpv_dna: Negative |
| GSM1607774 | hpv_dna: Negative |
| GSM1607775 | hpv_dna: Negative |
| GSM1607776 | hpv_dna: Negative |
| GSM1607777 | hpv_dna: Negative |
| GSM1607778 | hpv_dna: Negative |
| GSM1607779 | hpv_dna: Negative |
| GSM1607780 | hpv_dna: Negative |
| GSM1607781 | hpv_dna: Negative |
| GSM1607782 | hpv_dna: Negative |
| GSM1607784 | hpv_dna: Negative |
| GSM1607785 | hpv_dna: Negative |
| GSM1607786 | hpv_dna: Negative |
| GSM1607787 | hpv_dna: Negative |
| GSM1607788 | hpv_dna: Negative |
| GSM1607789 | hpv_dna: Negative |
| GSM1607790 | hpv_dna: Negative |
| GSM1607791 | hpv_dna: Negative |
| GSM1607792 | hpv_dna: Negative |
| GSM1607793 | hpv_dna: Negative |
| GSM1607794 | hpv_dna: Negative |
| GSM1607795 | hpv_dna: Negative |
| GSM1607797 | hpv_dna: Negative |
| GSM1607798 | hpv_dna: Negative |
| GSM1607799 | hpv_dna: Negative |
| GSM1607801 | hpv_dna: Negative |
| GSM1607802 | hpv_dna: Negative |
| GSM1607803 | hpv_dna: Negative |
| GSM1607804 | hpv_dna: Negative |
| GSM1607805 | hpv_dna: Negative |
| GSM1607806 | hpv_dna: Negative |
| GSM1607807 | hpv_dna: Negative |

| | |
|---|---|
| GSM1607808 | hpv_dna: Negative |
| GSM1607809 | hpv_dna: Negative |
| GSM1607810 | hpv_dna: Negative |
| GSM1607811 | hpv_dna: Negative |
| GSM1607812 | hpv_dna: Negative |
| GSM1607813 | hpv_dna: Negative |
| GSM1607814 | hpv_dna: Negative |
| GSM1607815 | hpv_dna: Negative |
| GSM1607816 | hpv_dna: Negative |
| GSM1607818 | hpv_dna: Negative |
| GSM1607819 | hpv_dna: Other HPV |
| GSM1607820 | hpv_dna: Negative |
| GSM1607823 | hpv_dna: Negative |
| GSM1607824 | hpv_dna: Negative |
| GSM1607825 | hpv_dna: Negative |
| GSM1607826 | hpv_dna: Negative |
| GSM1607827 | hpv_dna: Negative |
| GSM1607828 | hpv_dna: Negative |
| GSM1607829 | hpv_dna: Negative |
| GSM1607830 | hpv_dna: Negative |
| GSM1607831 | hpv_dna: Negative |
| GSM1607832 | hpv_dna: Negative |
| GSM1607833 | hpv_dna: Negative |
| GSM1607835 | hpv_dna: Negative |
| GSM1607836 | hpv_dna: Negative |
| GSM1607837 | hpv_dna: Negative |
| GSM1607839 | hpv_dna: Negative |
| GSM1607840 | hpv_dna: Negative |
| GSM1607841 | hpv_dna: Negative |
| GSM1607842 | hpv_dna: Negative |
| GSM1607843 | hpv_dna: Negative |
| GSM1607844 | hpv_dna: Negative |
| GSM1607845 | hpv_dna: Negative |
| GSM1607846 | hpv_dna: Negative |
| GSM1607847 | hpv_dna: Negative |
| GSM1607848 | hpv_dna: Negative |
| GSM1607849 | hpv_dna: Negative |
| GSM1607850 | hpv_dna: Negative |
| GSM1607851 | hpv_dna: Negative |
| GSM1607852 | hpv_dna: Negative |
| GSM1607853 | hpv_dna: Negative |
| GSM1607854 | hpv_dna: Negative |
| GSM1607855 | hpv_dna: Negative |
| GSM1607856 | hpv_dna: Negative |

| | |
|---|---|
| GSM1607857 | hpv_dna: Negative |
| GSM1607859 | hpv_dna: Negative |
| GSM1607860 | hpv_dna: Negative |
| GSM1607861 | hpv_dna: Negative |
| GSM1607862 | hpv_dna: Negative |
| GSM1607863 | hpv_dna: Negative |
| GSM1607864 | hpv_dna: Negative |
| GSM1607865 | hpv_dna: Negative |
| GSM1607866 | hpv_dna: Negative |
| GSM1607867 | hpv_dna: Negative |
| GSM1607869 | hpv_dna: Negative |
| GSM1607871 | hpv_dna: Negative |
| GSM1607872 | hpv_dna: Negative |
| GSM1607873 | hpv_dna: Negative |
| GSM1607874 | hpv_dna: Negative |
| GSM1607875 | hpv_dna: Negative |
| GSM1607879 | hpv_dna: Other HPV |
| GSM1607882 | hpv_dna: Negative |
| GSM1607883 | hpv_dna: Negative |
| GSM1607884 | hpv_dna: Negative |
| GSM1607885 | hpv_dna: Negative |
| GSM1607886 | hpv_dna: Negative |
| GSM1607888 | hpv_dna: Negative |
| GSM1607889 | hpv_dna: Negative |
| GSM1607890 | hpv_dna: Negative |
| GSM1607891 | hpv_dna: Negative |
| GSM1607892 | hpv_dna: Negative |
| GSM1607894 | hpv_dna: Negative |
| GSM1607895 | hpv_dna: Negative |
| GSM1607896 | hpv_dna: Negative |
| GSM1607897 | hpv_dna: Negative |
| GSM1607898 | hpv_dna: Negative |
| GSM1607901 | hpv_dna: Negative |
| GSM1607906 | hpv_dna: Negative |
| GSM1607907 | hpv_dna: Negative |
| GSM1607908 | hpv_dna: Negative |
| GSM1607909 | hpv_dna: Negative |
| GSM1607911 | hpv_dna: Negative |
| GSM1607912 | hpv_dna: Negative |
| GSM1607914 | hpv_dna: Other HPV |
| GSM1607915 | hpv_dna: Negative |
| GSM1607916 | hpv_dna: Negative |
| GSM1607917 | hpv_dna: Negative |
| GSM1607918 | hpv_dna: Negative |

| | |
|---|---|
| GSM1607919 | hpv_dna: Negative |
| GSM1607920 | hpv_dna: Negative |
| GSM1607921 | hpv_dna: Negative |
| GSM1607922 | hpv_dna: Negative |
| GSM1607923 | hpv_dna: Negative |
| GSM1607924 | hpv_dna: Negative |
| GSM1607925 | hpv_dna: Negative |
| GSM1607926 | hpv_dna: Negative |
| GSM1607927 | hpv_dna: Negative |
| GSM1607928 | hpv_dna: Negative |
| GSM1607929 | hpv_dna: Negative |
| GSM1607930 | hpv_dna: Negative |
| GSM1607932 | hpv_dna: Negative |
| GSM1607934 | hpv_dna: Negative |
| GSM1607935 | hpv_dna: Negative |
| GSM1607936 | hpv_dna: Negative |
| GSM1607937 | hpv_dna: Negative |
| GSM1607938 | hpv_dna: Negative |
| GSM1607939 | hpv_dna: Other HPV |
| GSM1607940 | hpv_dna: Negative |
| GSM1607943 | hpv_dna: Negative |
| GSM1607944 | hpv_dna: Negative |
| GSM1607945 | hpv_dna: Negative |
| GSM1607946 | hpv_dna: Negative |
| GSM1607947 | hpv_dna: Negative |
| GSM1607948 | hpv_dna: Negative |
| GSM1607949 | hpv_dna: Negative |
| GSM1607950 | hpv_dna: Negative |
| GSM1607951 | hpv_dna: Negative |
| GSM1607952 | hpv_dna: Negative |
| GSM1607953 | hpv_dna: Negative |

**Supplementary Table S2.** DNA damage response gene sets

| Gene symbol | NCBI (Entrez) Gene ID | DNA damage response type |
|---|---|---|
| RAD50 | 10111 | Homologous recombination |
| POLD3 | 10714 | Homologous recombination |
| EME1 | 146956 | Homologous recombination |
| RAD54B | 25788 | Homologous recombination |
| RPA4 | 29935 | Homologous recombination |
| MRE11 | 4361 | Homologous recombination |
| NBN | 4683 | Homologous recombination |

| | | |
|---|---|---|
| POLD1 | 5424 | Homologous recombination |
| POLD2 | 5425 | Homologous recombination |
| POLD4 | 57804 | Homologous recombination |
| RAD51 | 5888 | Homologous recombination |
| RAD51C | 5889 | Homologous recombination |
| RAD51B | 5890 | Homologous recombination |
| RAD51D | 5892 | Homologous recombination |
| RAD52 | 5893 | Homologous recombination |
| RPA1 | 6117 | Homologous recombination |
| RPA2 | 6118 | Homologous recombination |
| RPA3 | 6119 | Homologous recombination |
| BLM | 641 | Homologous recombination |
| SSBP1 | 6742 | Homologous recombination |
| BRCA2 | 675 | Homologous recombination |
| TOP3A | 7156 | Homologous recombination |
| XRCC2 | 7516 | Homologous recombination |
| XRCC3 | 7517 | Homologous recombination |
| SEM1 | 7979 | Homologous recombination |
| MUS81 | 80198 | Homologous recombination |
| RAD54L | 8438 | Homologous recombination |
| TOP3B | 8940 | Homologous recombination |
| BRCA1 | 672 | Homologous recombination |
| RFC2 | 5982 | Homologous recombination |
| PALB2 | 79728 | Homologous recombination |
| RBBP8 | 5932 | Homologous recombination |
| RAD51AP1 | 10635 | Homologous recombination |
| RFC5 | 5985 | Homologous recombination |
| RAD1 | 5810 | Homologous recombination |
| RFC3 | 5983 | Homologous recombination |
| HUS1 | 3364 | Homologous recombination |
| BRIP1 | 83990 | Homologous recombination |
| DNA2 | 1763 | Homologous recombination |
| BARD1 | 580 | Homologous recombination |
| ATM | 472 | Homologous recombination |
| CHEK1 | 1111 | Homologous recombination |
| RAD9B | 144715 | Homologous recombination |
| RAD17 | 5884 | Homologous recombination |
| TOPBP1 | 11073 | Homologous recombination |
| RFC4 | 5984 | Homologous recombination |
| ATRIP | 84126 | Homologous recombination |
| WRN | 7486 | Homologous recombination |
| RHNO1 | 83695 | Homologous recombination |
| RAD9A | 5883 | Homologous recombination |
| KAT5 | 10524 | Homologous recombination |

| | | |
|---|---|---|
| EXO1 | 9156 | Homologous recombination |
| ATR | 545 | Homologous recombination |
| RMI2 | 116028 | Homologous recombination |
| RMI1 | 80010 | Homologous recombination |
| RAD50 | 10111 | Non-homologous end joining |
| DNTT | 1791 | Non-homologous end joining |
| FEN1 | 2237 | Non-homologous end joining |
| XRCC6 | 2547 | Non-homologous end joining |
| POLL | 27343 | Non-homologous end joining |
| POLM | 27434 | Non-homologous end joining |
| LIG4 | 3981 | Non-homologous end joining |
| MRE11 | 4361 | Non-homologous end joining |
| PRKDC | 5591 | Non-homologous end joining |
| DCLRE1C | 64421 | Non-homologous end joining |
| XRCC4 | 7518 | Non-homologous end joining |
| XRCC5 | 7520 | Non-homologous end joining |
| NHEJ1 | 79840 | Non-homologous end joining |
| POLD1 | 5424 | Mismatch repair |
| MLH1 | 4292 | Mismatch repair |
| POLD3 | 10714 | Mismatch repair |
| MSH2 | 4436 | Mismatch repair |
| LIG1 | 3978 | Mismatch repair |
| RPA3 | 6119 | Mismatch repair |
| POLD2 | 5425 | Mismatch repair |
| MSH3 | 4437 | Mismatch repair |
| MSH6 | 2956 | Mismatch repair |
| RPA2 | 6118 | Mismatch repair |
| PMS2 | 5395 | Mismatch repair |
| RPA1 | 6117 | Mismatch repair |
| PCNA | 5111 | Mismatch repair |
| EXO1 | 9156 | Mismatch repair |
| POLD4 | 57804 | Mismatch repair |
| MLH3 | 27030 | Mismatch repair |
| RPA4 | 29935 | Mismatch repair |
| RFC1 | 5981 | Mismatch repair |
| RFC2 | 5982 | Mismatch repair |
| RFC3 | 5983 | Mismatch repair |
| RFC4 | 5984 | Mismatch repair |
| RFC5 | 5985 | Mismatch repair |
| SSBP1 | 6742 | Mismatch repair |
| CDK7 | 1022 | Nucleotide excision repair |
| CETN2 | 1069 | Nucleotide excision repair |
| POLD3 | 10714 | Nucleotide excision repair |
| ERCC8 | 1161 | Nucleotide excision repair |

| | | |
|---|---|---|
| DDB1 | 1642 | Nucleotide excision repair |
| DDB2 | 1643 | Nucleotide excision repair |
| ERCC1 | 2067 | Nucleotide excision repair |
| ERCC2 | 2068 | Nucleotide excision repair |
| ERCC3 | 2071 | Nucleotide excision repair |
| ERCC4 | 2072 | Nucleotide excision repair |
| ERCC5 | 2073 | Nucleotide excision repair |
| ERCC6 | 2074 | Nucleotide excision repair |
| GTF2H1 | 2965 | Nucleotide excision repair |
| GTF2H2 | 2966 | Nucleotide excision repair |
| GTF2H3 | 2967 | Nucleotide excision repair |
| GTF2H4 | 2968 | Nucleotide excision repair |
| RPA4 | 29935 | Nucleotide excision repair |
| LIG1 | 3978 | Nucleotide excision repair |
| GTF2H5 | 404672 | Nucleotide excision repair |
| MNAT1 | 4331 | Nucleotide excision repair |
| PCNA | 5111 | Nucleotide excision repair |
| POLE3 | 54107 | Nucleotide excision repair |
| POLD1 | 5424 | Nucleotide excision repair |
| POLD2 | 5425 | Nucleotide excision repair |
| POLE | 5426 | Nucleotide excision repair |
| POLE2 | 5427 | Nucleotide excision repair |
| POLE4 | 56655 | Nucleotide excision repair |
| POLD4 | 57804 | Nucleotide excision repair |
| RAD23A | 5886 | Nucleotide excision repair |
| RAD23B | 5887 | Nucleotide excision repair |
| RFC1 | 5981 | Nucleotide excision repair |
| RFC2 | 5982 | Nucleotide excision repair |
| RFC3 | 5983 | Nucleotide excision repair |
| RFC4 | 5984 | Nucleotide excision repair |
| RFC5 | 5985 | Nucleotide excision repair |
| RPA1 | 6117 | Nucleotide excision repair |
| RPA2 | 6118 | Nucleotide excision repair |
| RPA3 | 6119 | Nucleotide excision repair |
| XPA | 7507 | Nucleotide excision repair |
| XPC | 7508 | Nucleotide excision repair |
| CUL4B | 8450 | Nucleotide excision repair |
| CUL4A | 8451 | Nucleotide excision repair |
| CCNH | 902 | Nucleotide excision repair |
| RBX1 | 9978 | Nucleotide excision repair |
| POLR2J | 5439 | Nucleotide excision repair |
| LIG3 | 3980 | Nucleotide excision repair |
| AQR | 9716 | Nucleotide excision repair |
| PIAS1 | 8554 | Nucleotide excision repair |

| | | |
|---|---|---|
| POLR2B | 5431 | Nucleotide excision repair |
| XRCC1 | 7515 | Nucleotide excision repair |
| ACTB | 60 | Nucleotide excision repair |
| XAB2 | 56949 | Nucleotide excision repair |
| PPIE | 10450 | Nucleotide excision repair |
| POLR2E | 5434 | Nucleotide excision repair |
| POLR2F | 5435 | Nucleotide excision repair |
| EP300 | 2033 | Nucleotide excision repair |
| YY1 | 7528 | Nucleotide excision repair |
| ACTR5 | 79913 | Nucleotide excision repair |
| POLR2C | 5432 | Nucleotide excision repair |
| UBE2I | 7329 | Nucleotide excision repair |
| POLR2I | 5438 | Nucleotide excision repair |
| TFPT | 29844 | Nucleotide excision repair |
| ELL | 8178 | Nucleotide excision repair |
| PRPF19 | 27339 | Nucleotide excision repair |
| COPS7A | 50813 | Nucleotide excision repair |
| ACTR8 | 93973 | Nucleotide excision repair |
| INO80D | 54891 | Nucleotide excision repair |
| INO80B | 83444 | Nucleotide excision repair |
| SUMO1 | 7341 | Nucleotide excision repair |
| COPS5 | 10987 | Nucleotide excision repair |
| POLK | 51426 | Nucleotide excision repair |
| USP45 | 85015 | Nucleotide excision repair |
| INO80 | 54617 | Nucleotide excision repair |
| PARP2 | 10038 | Nucleotide excision repair |
| CHD1L | 9557 | Nucleotide excision repair |
| PIAS3 | 10401 | Nucleotide excision repair |
| ACTL6A | 86 | Nucleotide excision repair |
| COPS4 | 51138 | Nucleotide excision repair |
| COPS3 | 8533 | Nucleotide excision repair |
| PARP1 | 142 | Nucleotide excision repair |
| RPS27A | 6233 | Nucleotide excision repair |
| POLR2D | 5433 | Nucleotide excision repair |
| COPS7B | 64708 | Nucleotide excision repair |
| POLR2K | 5440 | Nucleotide excision repair |
| UBC | 7316 | Nucleotide excision repair |
| INO80C | 125476 | Nucleotide excision repair |
| RNF111 | 54778 | Nucleotide excision repair |
| POLR2H | 5437 | Nucleotide excision repair |
| UVSSA | 57654 | Nucleotide excision repair |
| COPS2 | 9318 | Nucleotide excision repair |
| POLR2G | 5436 | Nucleotide excision repair |
| COPS6 | 10980 | Nucleotide excision repair |

| | | |
|---|---|---|
| UBE2V2 | 7336 | Nucleotide excision repair |
| INO80E | 283899 | Nucleotide excision repair |
| GPS1 | 2873 | Nucleotide excision repair |
| UBB | 7314 | Nucleotide excision repair |
| NFRKB | 4798 | Nucleotide excision repair |
| RUVBL1 | 8607 | Nucleotide excision repair |
| POLR2L | 5441 | Nucleotide excision repair |
| UBE2N | 7334 | Nucleotide excision repair |
| POLR2A | 5430 | Nucleotide excision repair |
| SUMO3 | 6612 | Nucleotide excision repair |
| USP7 | 7874 | Nucleotide excision repair |
| TCEA1 | 6917 | Nucleotide excision repair |
| MCRS1 | 10445 | Nucleotide excision repair |
| SUMO2 | 6613 | Nucleotide excision repair |
| COPS8 | 10920 | Nucleotide excision repair |
| ZNF830 | 91603 | Nucleotide excision repair |
| HMGN1 | 3150 | Nucleotide excision repair |
| UBA52 | 7311 | Nucleotide excision repair |
| ISY1 | 57461 | Nucleotide excision repair |
| PARP2 | 10038 | Base excision repair |
| PARP3 | 10039 | Base excision repair |
| HMGB1P1 | 10357 | Base excision repair |
| POLD3 | 10714 | Base excision repair |
| PARP1 | 142 | Base excision repair |
| PARP4 | 143 | Base excision repair |
| FEN1 | 2237 | Base excision repair |
| SMUG1 | 23583 | Base excision repair |
| NEIL2 | 252969 | Base excision repair |
| APEX2 | 27301 | Base excision repair |
| POLL | 27343 | Base excision repair |
| HMGB1 | 3146 | Base excision repair |
| APEX1 | 328 | Base excision repair |
| LIG1 | 3978 | Base excision repair |
| LIG3 | 3980 | Base excision repair |
| MPG | 4350 | Base excision repair |
| MUTYH | 4595 | Base excision repair |
| NTHL1 | 4913 | Base excision repair |
| OGG1 | 4968 | Base excision repair |
| PCNA | 5111 | Base excision repair |
| POLE3 | 54107 | Base excision repair |
| POLB | 5423 | Base excision repair |
| POLD1 | 5424 | Base excision repair |
| POLD2 | 5425 | Base excision repair |
| POLE | 5426 | Base excision repair |

| | | |
|---|---|---|
| POLE2 | 5427 | Base excision repair |
| NEIL3 | 55247 | Base excision repair |
| POLE4 | 56655 | Base excision repair |
| POLD4 | 57804 | Base excision repair |
| HMGB1P40 | 100506595 | Base excision repair |
| TDG | 6996 | Base excision repair |
| UNG | 7374 | Base excision repair |
| XRCC1 | 7515 | Base excision repair |
| NEIL1 | 79661 | Base excision repair |
| MBD4 | 8930 | Base excision repair |
| RFC1 | 5981 | Base excision repair |
| PNKP | 11284 | Base excision repair |
| RFC2 | 5982 | Base excision repair |
| TINF2 | 26277 | Base excision repair |
| ACD | 65057 | Base excision repair |
| H2AZ2 | 94239 | Base excision repair |
| RPA3 | 6119 | Base excision repair |
| RFC5 | 5985 | Base excision repair |
| ADPRS | 54936 | Base excision repair |
| RPA2 | 6118 | Base excision repair |
| H2BC11 | 8970 | Base excision repair |
| POT1 | 25913 | Base excision repair |
| RPA1 | 6117 | Base excision repair |
| TERF2 | 7014 | Base excision repair |
| RFC3 | 5983 | Base excision repair |
| H2BC1 | 255626 | Base excision repair |
| TERF1 | 7013 | Base excision repair |
| H2BC5 | 3017 | Base excision repair |
| H4C8 | 8365 | Base excision repair |
| RFC4 | 5984 | Base excision repair |
| H2AZ1 | 3015 | Base excision repair |
| TERF2IP | 54386 | Base excision repair |
| H3-4 | 8290 | Base excision repair |
| H2AC6 | 8334 | Base excision repair |
| H2BC4 | 8347 | Base excision repair |
| H2AC20 | 8338 | Base excision repair |
| H2BC21 | 8349 | Base excision repair |
| H2BC13 | 8340 | Base excision repair |
| H2AX | 3014 | Base excision repair |
| H2AC7 | 3013 | Base excision repair |
| H2BU1 | 128312 | Base excision repair |
| H4C3 | 8364 | Base excision repair |
| H4C11 | 8363 | Base excision repair |
| H4-16 | 121504 | Base excision repair |

| | | |
|---|---|---|
| H2BC12 | 85236 | Base excision repair |
| H2AC18 | 8337 | Base excision repair |
| PARG | 8505 | Base excision repair |
| H2BC15 | 8341 | Base excision repair |
| H2BS1 | 54145 | Base excision repair |
| H2AJ | 55766 | Base excision repair |
| H4C15 | 554313 | Base excision repair |
| H4C14 | 8370 | Base excision repair |
| H2AC19 | 723790 | Base excision repair |
| H4C12 | 8362 | Base excision repair |
| H2BC14 | 8342 | Base excision repair |
| H2BC8 | 8339 | Base excision repair |
| H2AB1 | 474382 | Base excision repair |
| H2BC6 | 8344 | Base excision repair |
| H4C6 | 8361 | Base excision repair |
| H2BC17 | 8348 | Base excision repair |
| H4C13 | 8368 | Base excision repair |
| H2BC9 | 8345 | Base excision repair |
| H4C9 | 8294 | Base excision repair |
| H2AC14 | 8331 | Base excision repair |
| H2BC3 | 3018 | Base excision repair |
| H4C5 | 8367 | Base excision repair |
| H2AC8 | 3012 | Base excision repair |
| H4C4 | 8360 | Base excision repair |
| H2BC7 | 8343 | Base excision repair |
| H2AC4 | 8335 | Base excision repair |
| H2BC10 | 8346 | Base excision repair |
| H4C1 | 8359 | Base excision repair |
| H4C2 | 8366 | Base excision repair |

**Supplementary Table S3.** Baseline clinicopathological and treatment characteristics in final discovery cohort.

| **Clinical and treatment related character** | **Whole cohort (n=364)** | **DDR_high/TM_low (n=53)** | **DDR_high/TM_high (n=311)** | ***P* value** |
|---|---|---|---|---|
| Sex | | | | **0.017*** |
| Female | 101 (27.7) | 7 (13.2) | 94 (30.2) | |
| Male | 263 (72.3) | 46 (86.8) | 217 (69.8) | |
| Grade | | | | 0.597 |
| G1 | 48 (13.2) | 7 (13.2) | 41 (13.2) | |
| G2 | 220 (60.4) | 33 (62.3) | 187 (60.1) | |

| | | | | |
|---|---|---|---|---|
| G3 | 84 (23.1) | 10 (18.9) | 74 (23.8) | |
| G4 | 2 (0.5) | 0 (0.0) | 2 (0.6) | |
| N/A | 10 (2.7) | 3 (5.7) | 7 (2.3) | |
| N stage | | | | 0.621 |
| N0 | 183 (50.3) | 28 (52.8) | 155 (49.8) | |
| N1 | 58 (15.9) | 5 (9.4) | 53 (17.0) | |
| N2 | 99 (27.2) | 17 (32.1) | 82 (26.4) | |
| N3 | 5 (1.4) | 1 (1.9) | 4 (1.3) | |
| N/A | 19 (5.2) | 2 (3.8) | 17 (5.5) | |
| T stage | | | | 0.454 |
| T1 | 21 (5.8) | 1 (1.9) | 20 (6.4) | |
| T2 | 92 (25.3) | 10 (18.9) | 82 (26.4) | |
| T3 | 99 (27.2) | 16 (30.2) | 83 (26.7) | |
| T4 | 139 (38.2) | 24 (45.3) | 115 (37.0) | |
| N/A | 13 (3.6) | 2 (3.8) | 11 (3.5) | |
| Stage | | | | 0.82 |
| I | 13 (3.6) | 1 (1.9) | 12 (3.9) | |
| II | 70 (19.2) | 8 (15.1) | 62 (19.9) | |
| III | 78 (21.4) | 11 (20.8) | 67 (21.5) | |
| IV | 190 (52.2) | 31 (58.5) | 159 (51.1) | |
| N/A | 13 (3.6) | 2 (3.8) | 11 (3.5) | |
| Age | | | | **0.01*** |
| <= 60 years | 164 (45.1) | 33 (62.3) | 131 (42.1) | |
| > 60 years | 200 (54.9) | 20 (37.7) | 180 (57.9) | |
| Alcohol | | | | 0.564 |
| NO | 125 (34.3) | 15 (28.3) | 110 (35.4) | |
| YES | 229 (62.9) | 36 (67.9) | 193 (62.1) | |
| N/A | 10 (2.7) | 2 (3.8) | 8 (2.6) | |
| Smoking | | | | 0.594 |
| NO | 129 (35.4) | 21 (39.6) | 108 (34.7) | |
| YES | 235 (64.6) | 32 (60.4) | 203 (65.3) | |
| ESTIMATE score | | | | **<0.001***** |
| ESTIMATE score-H(TM-H) | 311 (85.4) | 0 (0.0) | 311 (100.0) | |
| ESTIMATE score-L(TM-L) | 53 (14.6) | 53 (100.0) | 0 (0.0) | |
| Lymphovascular invasion | | | | 0.406 |
| NO | 164 (45.1) | 20 (37.7) | 144 (46.3) | |
| YES | 89 (24.5) | 13 (24.5) | 76 (24.4) | |
| N/A | 111 (30.5) | 20 (37.7) | 91 (29.3) | |
| Margin status | | | | 0.702 |
| Close | 40 (12.1) | 4 (8.9) | 36 (12.6) | |
| Negative | 247 (74.8) | 34 (75.6) | 213 (74.7) | |
| Positive | 43 (13.0) | 7 (15.6) | 36 (12.6) | |
| Perineural invasion | | | | 0.626 |

| | | | | |
|---|---|---|---|---|
| NO | 140 (38.5) | 20 (37.7) | 120 (38.6) | |
| YES | 119 (32.7) | 15 (28.3) | 104 (33.4) | |
| N/A | 105 (28.8) | 18 (34.0) | 87 (28.0) | |
| Pathological nodal extracapsular spread | | | | 0.254 |
| Gross extension | 28 (7.7) | 7 (13.2) | 21 (6.8) | |
| Microscopic extension | 54 (14.8) | 6 (11.3) | 48 (15.4) | |
| No extranodal extension | 176 (48.4) | 22 (41.5) | 154 (49.5) | |
| N/A | 106 (29.1) | 18 (34.0) | 88 (28.3) | |
| Neoadjuvant treatment | | | | 0.603 |
| No | 357 (98.1) | 51 (96.2) | 306 (98.4) | |
| Yes | 7 (1.9) | 2 (3.8) | 5 (1.6) | |
| Radiation therapy | | | | 0.98 |
| NO | 116 (31.9) | 17 (32.1) | 99 (31.8) | |
| YES | 196 (53.8) | 28 (52.8) | 168 (54.0) | |
| N/A | 52 (14.3) | 8 (15.1) | 44 (14.1) | |
| Additional pharmaceutical therapy | | | | 0.764 |
| NO | 47 (12.9) | 8 (15.1) | 39 (12.5) | |
| YES | 35 (9.6) | 6 (11.3) | 29 (9.3) | |
| N/A | 282 (77.5) | 39 (73.6) | 243 (78.1) | |
| Additional radiation therapy | | | | 0.222 |
| NO | 61 (16.8) | 13 (24.5) | 48 (15.4) | |
| YES | 22 (6.0) | 2 (3.8) | 20 (6.4) | |
| N/A | 281 (77.2) | 38 (71.7) | 243 (78.1) | |
| Anatomic neoplasm subdivision | | | | 0.421 |
| Hypopharynx | 5 (1.4) | 2 (3.8) | 3 (1.0) | |
| Larynx | 98 (26.9) | 16 (30.2) | 82 (26.4) | |
| Lip | 2 (0.5) | 0 (0.0) | 2 (0.6) | |
| Oral Cavity | 231 (63.5) | 30 (56.6) | 201 (64.6) | |
| Oropharynx | 28 (7.7) | 5 (9.4) | 23 (7.4) | |

Note: **P* value<0.05; ***P* value<0.01; ****P* value<0.001

Abbreviations: HR, hazard ratio; CI, confidence interval; DDR-high, High DNA repair level in Transcriptome; TM-high/-low, tumor microenvironment score high/low in Transcriptome, respectively. N/A, not available.

Figure S1. The top 100 hub genes selected in blue (A) ,green (B) and red (C) modules by protein-to-protein network method.

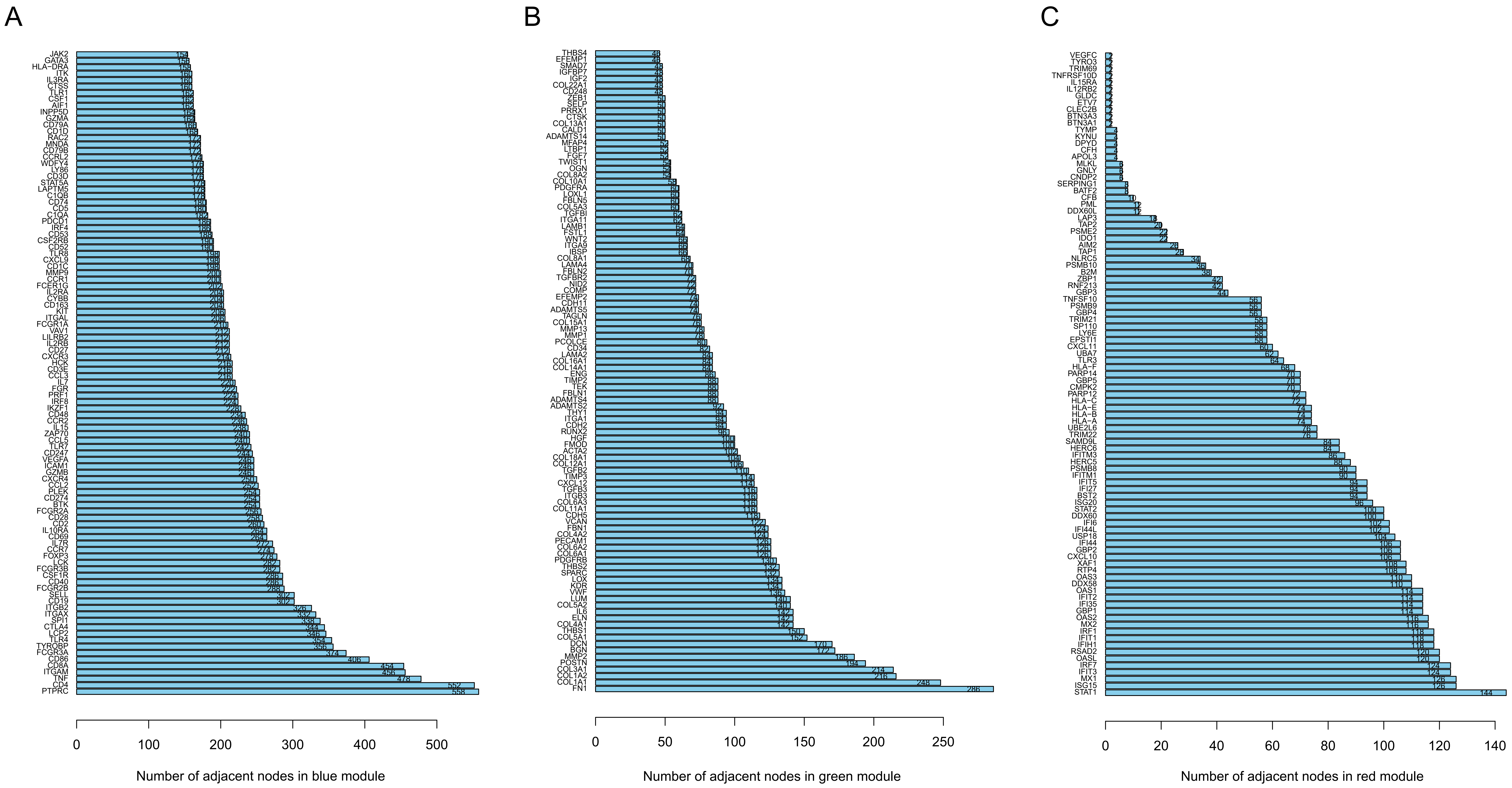

Figure S2. Drug sensitivity under hub genes expression in Cancer Therapeutics Response Portal (CTRP) via GSCALite platform. EGFR was listed here as an internal control group to check the predictability of drug sensitivity.

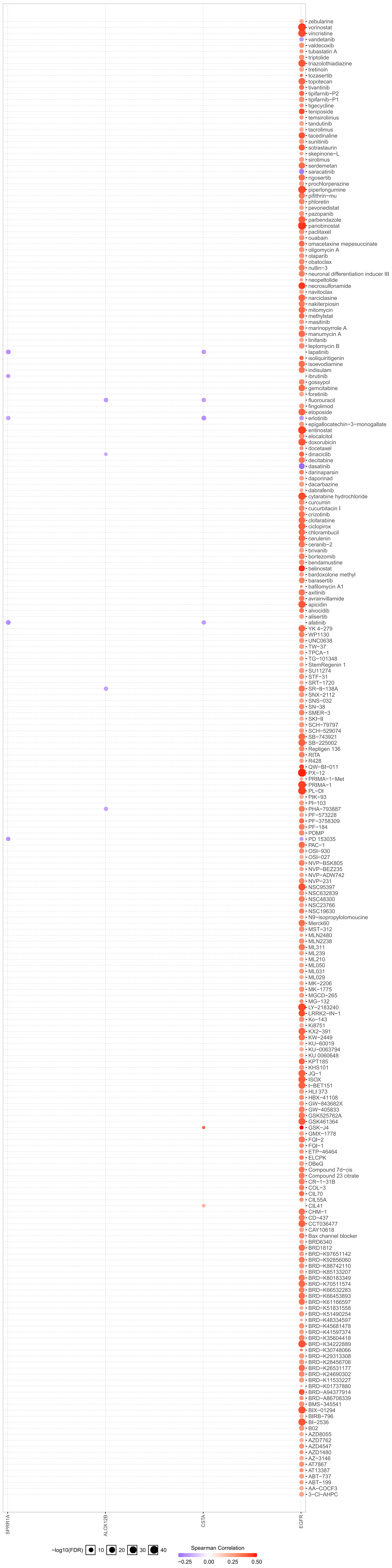

Figure S3. Drug sensitivity under hub genes expression in Genomics of Drug Sensitivity in Cancer (GDSC) via GSCALite platform. EGFR was listed here as an internal control group to check the predictability of drug sensitivity.

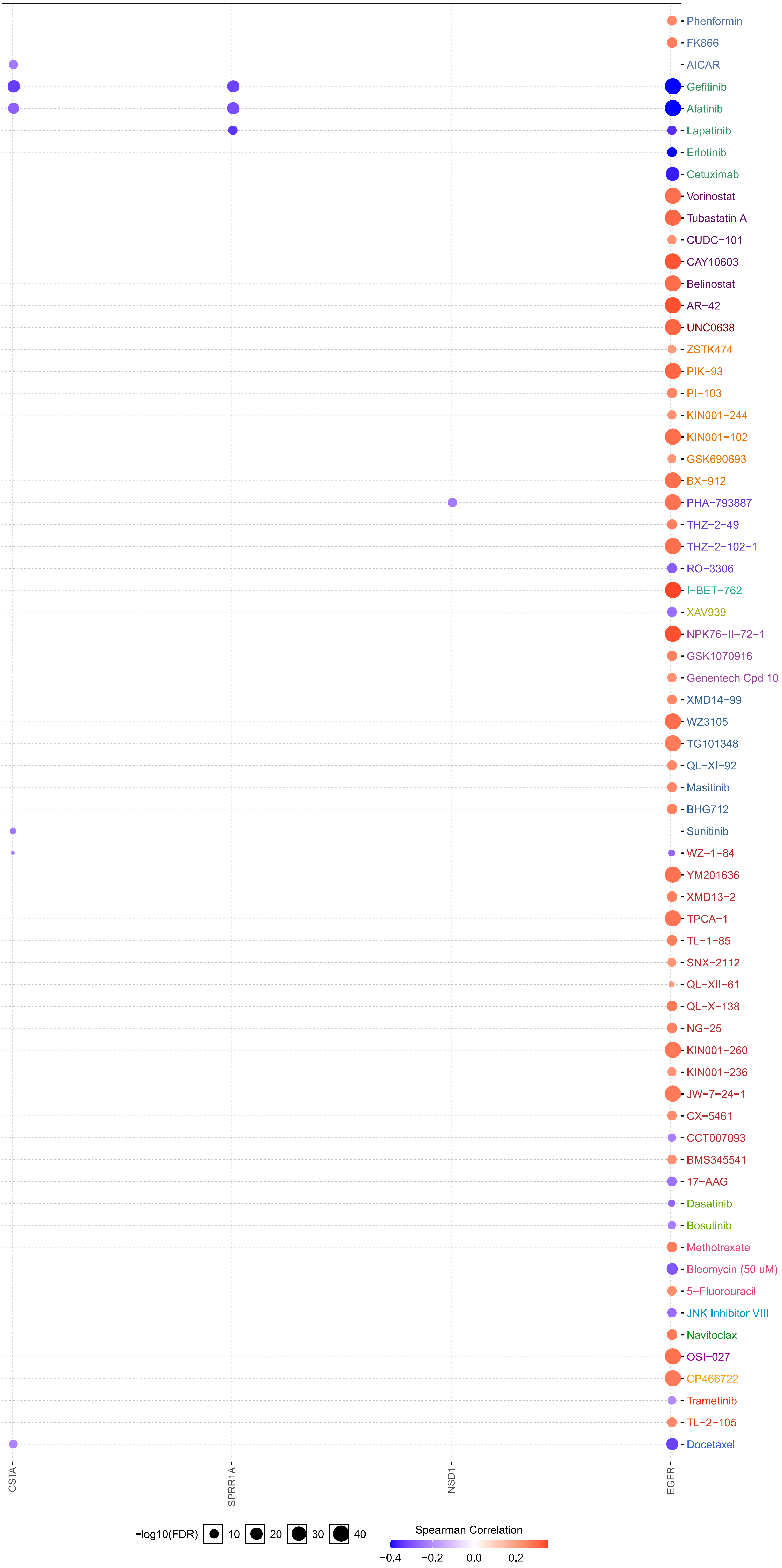

Supplement: Supplementary file 1 — Additional file 1. [file 12885_2022_9722_MOESM1_ESM.pdf]
